# Different scaling of linear models and deep learning in UKBiobank brain images versus machine-learning datasets

**DOI:** 10.1038/s41467-020-18037-z

**Published:** 2020-08-25

**Authors:** Marc-Andre Schulz, B. T. Thomas Yeo, Joshua T. Vogelstein, Janaina Mourao-Miranada, Jakob N. Kather, Konrad Kording, Blake Richards, Danilo Bzdok

**Affiliations:** 1 Department of Psychiatry, Psychotherapy, and Psychosomatics, Rheinisch-Westfälische Technische Hochschule (RWTH), Aachen University, Aachen, Germany; 2 Department of Electrical and Computer Engineering, National University of Singapore, Singapore, Singapore; 3 Centre for Sleep and Cognition (CSC) and Centre for Translational Magnetic Resonance Research (TMR), National University of Singapore, Singapore, Singapore; 4 N.1 Institute for Health and Institute for Digital Medicine (WisDM), National University of Singapore, Singapore, Singapore; 5 Department of Biomedical Engineering, Institute for Computational Medicine, Johns Hopkins University, Baltimore, Maryland USA; 6 Kavli Neuroscience Discovery Institute, Johns Hopkins University, Baltimore, Maryland USA; 7 Max Planck University College London Centre for Computational Psychiatry and Ageing Research, University College London, London, UK; 8 Centre for Medical Image Computing, Department of Computer Science, University College London, London, UK; 9 Department of Medicine III, University Hospital RWTH Aachen, Aachen, Germany; 10 German Cancer Consortium (DKTK), German Cancer Research Center (DKFZ), Heidelberg, Germany; 11 Applied Tumor Immunity, German Cancer Research Center (DKFZ), Heidelberg, Germany; 12 Department of Neuroscience and Department of Bioengineering, University of Pennsylvania, Philadelphia, Pennsylvania USA; 13 Department of Neurology and Neurosurgery, McGill University, Montréal, Québec Canada; 14 School of Computer Science, McGill University, Montréal, Québec Canada; 15 Canadian Institute for Advanced Research, Toronto, Ontario Canada; 16 Mila - Quebec Artificial Intelligence Institute, Montréal, Québec Canada; 17 Neurospin, Commissariat à l’Energie Atomique (CEA) Saclay, Gif-sur-Yvette, France; 18 Parietal Team, Institut National de Recherche en Informatique et en Automatique (INRIA), Gif-sur-Yvette, France; 19 Faculty of Medicine, Department of Biomedical Engineering, McConnell Brain imaging Centre, Montreal Neurological Institute (MNI), McGill University, Montreal, Québec Canada

**Keywords:** Neural decoding, Genetic databases

## Abstract

Recently, deep learning has unlocked unprecedented success in various domains, especially using images, text, and speech. However, deep learning is only beneficial if the data have nonlinear relationships and if they are exploitable at available sample sizes. We systematically profiled the performance of deep, kernel, and linear models as a function of sample size on UKBiobank brain images against established machine learning references. On MNIST and Zalando Fashion, prediction accuracy consistently improves when escalating from linear models to shallow-nonlinear models, and further improves with deep-nonlinear models. In contrast, using structural or functional brain scans, simple linear models perform on par with more complex, highly parameterized models in age/sex prediction across increasing sample sizes. In sum, linear models keep improving as the sample size approaches ~10,000 subjects. Yet, nonlinearities for predicting common phenotypes from typical brain scans remain largely inaccessible to the examined kernel and deep learning methods.

## Abstract (web summary)

Schulz *et al*. systematically benchmark performance scaling with increasingly sophisticated prediction algorithms and with increasing sample size in reference machine-learning and biomedical datasets. Complicated nonlinear intervariable relationships remain largely inaccessible for predicting key phenotypes from typical brain scans.

## Introduction

Following genetics and other biological domains, imaging neuroscience recently started to become a big data science. The brain sciences have been proposed to be one of the most data-rich medical specialties^1^ due to amassing high-resolution imaging data.

Several data collection initiatives stand out in the brain-imaging landscape^2,3^, including the Human Connectome Project (HCP), the UKBiobank (UKBB) imaging study, and the Enhancing NeuroImaging Genetics through Meta-Analysis (ENIGMA) consortium. The UKBB is today perhaps the most compelling, as this resource includes genetic profiling and an extensive variety of phenotyping descriptors. The data aggregation set out around 2006 to gather genetic and environmental data of ~500,000 volunteers and is currently the world’s largest biomedical dataset. In 2014, UKBB launched its brain-imaging extension, aiming to gather several magnetic resonance imaging (MRI) modalities of ~100,000 subjects by 2022^4^. UKBB is specifically designed for prospective population epidemiology. Instead, the ambition of HCP lies in functional and anatomical connectivity in healthy subjects, whereas ENIGMA places a premium on genetic profiling in combination with brain scanning in psychiatric and neurological disease. The creation, curation, and collaboration of large-scale brain-imaging datasets with thousands of subjects promises to enable more advanced quantitative analytics than are currently the norm.

An important benefit of such large-scale data collections is that they may allow for more expressive models that could more powerfully isolate and describe phenomena in the brain—models that can capture complicated nonlinear interactions dormant in commonly analyzed, now abundant brain scans. Spurred by increasing data availability, analysis of brain-imaging data is more and more endorsing sophisticated machine learning algorithms^5–7^. A core step in  such data analysis approaches has always been the identification of the most relevant variables to be included and how these candidate variables should be encoded or built into newly designed features, often called “manual feature engineering.”

Linear models have long dominated data analysis, as complex transformations into rich high-dimensional spaces were historically computationally infeasible^5,8,9^. Towards the end of the twentieth century, kernel embeddings^10^ were devised to efficiently map data to rich high-dimensional spaces in a computationally efficient manner. Kernel methods can perform data analysis within an enriched, potentially infinitely dimensional representation of the original input variables. This extension of many classical linear methods towards capturing more complicated nonlinear patterns in data enabled enhanced prediction accuracy in a large variety of applications, including many areas of biomedicine.

“Preprogrammed” kernel methods operating on predefined similarity functions, in turn, have recently been superseded by the renaissance of artificial neural networks under the umbrella term “deep learning^11^.” One key aspect of this even more flexible class of algorithms is the cascade of successive nonlinear transformations from the input variables. A deep neural network (DNN) automatically learns to combine image pixels into basic shapes such as circles or edges, which get combined by further transformation layers into objects such as furniture, which eventually compose whole scenes and other abstract concepts such as a kitchen, represented in the highest layers. Going beyond kernel methods, deep methods have enabled “automatic feature engineering” and even richer representations and abstractions of patterns in data. In a sense, deep learning methods can be thought of as kernel methods that also learn the kernel^12^.

This upgrade has unlocked unprecedented prediction success in a number of application domains, especially those involving the processing of natural images, text, or speech data—areas where DNNs can leverage some of their strengths: compositional representations and a hardcoded assumption of translation invariance. Whether deep learning will be equally successful in images from brain scanning, specifically in predicting phenotypes from structural and functional MRI (sMRI/fMRI), remains yet unclear^13^. An impartial evaluation of deep learning in brain imaging is urgently required. However, deep learning models are highly flexible and new varieties are constantly developed. This rank growth of deep learning models makes it almost impossible to comprehensively benchmark deep learning models, one-by-one, for brain imaging. Here we attempt to address this need by first principles. Our study brings  into sharp focus the precondition that is most likely to limit the success of deep learning on brain-imaging data: the extent to which nonlinear relationships in brain images are exploitable for phenotype prediction at currently available sample sizes.

In particular, an ingredient in the success of deep learning for image processing have been convolution operations, which introduce the additional assumption of translational invariance^11^. To use a geography analogy, representational features of traffic jams could be detected from satellite images by convolution layers no matter in which city the traffic jams are located and irrespective of their extent and form. These traffic events can then be picked up by higher-level convolution layers into state, country, and then continent summaries of global traffic patterns (i.e., compositionality), ignoring where the traffic information was aggregated from. This propagation would be made possible, because convolution layers assume that if image information is useful for quantifying traffic in one location, the same features will also be useful in other distant locations (i.e., translational invariance). However, despite the success of deep learning for many image-processing applications, it is still unclear to what extent MRI brain scans yield nonlinear structure exploitable for phenotype prediction that requires both compositionality and translational invariance. Are there higher-level features of an MRI scan that are composed of lower-level features in a nonlinear manner? Can phenotype prediction benefit from capturing irregular anatomical shapes such as those of brain ventricles? Do the same informative features appear in any part of an MRI image? If not, then the performance improvements that deep convolutional neural networks achieve in some image-processing domains may not directly port over to the analysis of MRI brain scans.

In short, from a historical perspective, kernel-based models have outperformed linear models in many applications. Deep learning models have again refined pattern extraction and thus further boosted prediction accuracy in structured data such as natural images. Our study systematically delineates the scaling behaviors of these three modeling regimes on brain-imaging data and compared to the performance profiles on standard machine learning datasets. Our quantitative findings provide some careful skepticism as to the question: do emerging large-scale brain-imaging datasets contain nonlinear information to better predict common phenotypes that can be exploited by currently available kernel and deep models?

## Results

### Rationale and summary of workflow

 The aim of our study was to assess how much the analysis of brain-imaging data can benefit from using nonlinear methods or even deep learning for predicting important demographic or lifestyle phenotypes. For results to be generalizable, we wished to shed light on the general types of information in brain-imaging data and whether those properties demand more complicated models making the most out of increasing sample size. Gaining such important intuitions would allow us to not only observe that a specific analytical approach works well on brain-imaging data, but provide indicators why that might be the case to allow for conclusions about broader classes of methods.

To this end, we evaluated how the prediction performance scales with increasing sample sizes for model classes with increasing prediction capacity and datasets of increasing prediction difficulty. We considered the achieved prediction performance as a function of the available sample size to obtain a principled empirical assessment of sample complexity (cf. “Methods”). Analyzing the empirical sample complexity allows for insight into the information content of data as perceived through the assumptions of a given model class. For example, linear models are blind to nonlinear patterns in data by construction. Nonlinear kernel methods assume that a certain type of nonlinear interaction (i.e., the kernel) is best suited to identify decision boundaries for accurate classification. Going another step up, DNNs also expect nonlinear interactions in the data, but, intuitively, this class of models learns the kernel rather than pre-assuming  this input variable expansion.

**Fig. 1:**
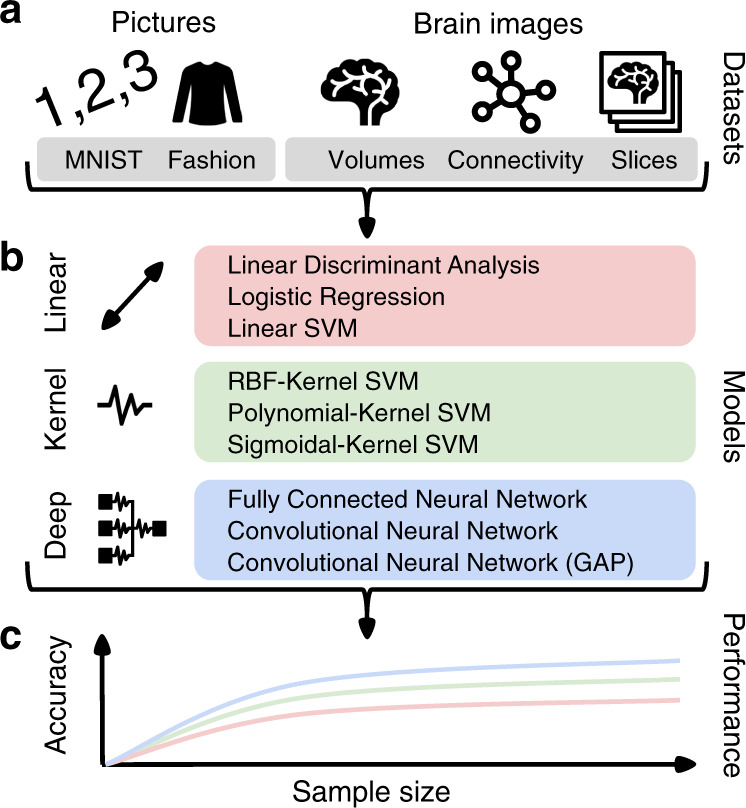
Workflow and experimental design. We directly compared the properties of machine learning datasets to brain-imaging datasets based on performance curves of three classes of predictive models. **a** Well-understood benchmark datasets from the machine learning community were elected to replicate previous results and serves as a reference point for the scaling behavior in relation to imaging neuroscience: the MNIST images were to be classified into different handwritten digits and Zalando’s Fashion images were to be classified into different types of clothing. Several representations of brain structure and function were obtained from the UKBiobank resource: region volumes and whole-brain slices from structural MRI, as well as functional connectivity strengths from resting-state functional MRI. Brain-image data were used to predict the subjects’ age and sex in 10 subgroups to match the ten target categories of MNIST and Fashion. **b** Prediction performance for each dataset was profiled using predictive models that have capacity for successively increasing predictive power: linear models (red tone), shallow-nonlinear kernel SVMs (green tone), and deep-nonlinear neural network algorithms (blue tones). **c** For each combination of dataset and model, we systematically varied the number of data points available for the training the model. The resulting empirical estimates of sample complexity allow to extrapolate conclusions to always larger sample sizes.

We employed three classes of learning algorithms to evaluate cross-validated phenotype prediction performance on reference datasets (Fig. 1) as follows: (a) classical (regularized) linear models are used for several decades in various empirical domains^14^, (b) kernel support vector machines (SVMs) that were among the most competitive approaches from the late 1990s to ~2010, and (c) common DNN algorithms that have now come to dominate areas where powerful empirical predictions are key and large amounts of structured (e.g., images) training data are available. For each model class, we chose three representative algorithms, attempting to cover the plurality of approaches in a given regime of quantitative analysis. For the class of linear methods, we elected linear discriminant analysis (LDA), logistic regression, and linear SVMs (without kernelization). For shallow-nonlinear models, we profiled extensions of linear models with the popular radial-basis-function (RBF), polynomial, and sigmoidal kernels. For deep-nonlinear models, we used fully connected and convolutional DNNs with and without global average pooling (GAP). We thus juxtaposed several analysis methods that recapitulate important periods in the recent data science evolution.

### Model performance on machine learning reference datasets

To verify that we can obtain empirical estimates of sample complexity differences between linear, kernel, and deep models, we initially examined two reference datasets that have been pervasively used in the machine learning community. This setup  did not only validate our approach to chart the scaling behavior of predictive performance with increasing sample size, but also established a point of comparison for performance differences for the three model classes when applied to brain-imaging data.

The MNIST dataset (Modified National Institute of Standards and Technology dataset^15^) is an important classical benchmark datasets in the machine learning community. This resource consists of greyscale images of handwritten digits with the digit value (0–9) to be classified from the raw pixel information. To quantitatively characterize the effects of a more challenging prediction goal, we also analyzed Zalando’s Fashion dataset^16^. The sometimes-called Fashion-MNIST dataset consists of grayscale images of fashion products, designed for classification of ten categories of clothing. This resource was created to pose a more difficult prediction problem than MNIST while retaining the same number of targets (i.e., 10 categories), dimensionality (i.e., 28 × 28 = 768 variables), and sample size (i.e., 70,000 images).

To make sure that our model implementations work as would be expected from the technical literature, we replicated several current-best prediction performances on the MNIST dataset. On the full dataset comprising 60,000 training observations, our logistic regression model achieved 91.32 ± 0.90% classification performance (out-of-sample prediction accuracy mean ± SD across 20 resampling iterations), which is very similar to earlier machine-learning studies (91.7% reported in ref. ^16^). The more modern SVMs with a RBF kernel (RBF-SVM) achieved 96.79 ± 0.65% accuracy (98.6% in ref. ^17^). Finally, standard convolutional DNN model achieved 99.03 ± 0.39% prediction accuracy—in line with the 98.9% reported by LeCun et al.^15^ based on a similar deep learning architecture. Our model performances thus replicated state-of-the-art classification accuracies that were reported in previous research in the technical communities.

**Fig. 2:**
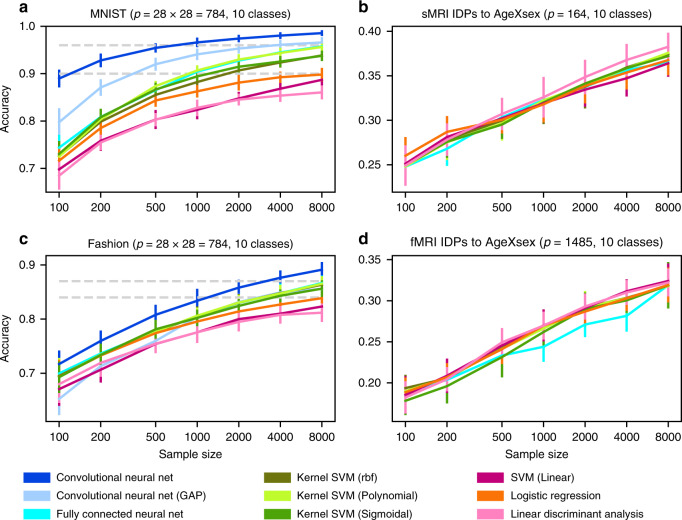
Classification performance gains with more powerful algorithms in two machine learning datasets. Shows performance scaling of prediction accuracy (*y* axis) as a function of increasing sample size (*x* axis) for generic linear models (red tones), kernel models (green tones), and deep neural network models (blue tones). All model performances are evaluated on the same independent test set. **a** In handwritten digit classification on the MNIST dataset, the classes of three linear, three kernel, and three deep models show distinct scaling behavior: linear models are outperformed by kernel models, which are, in turn, outperformed by deep models. The prediction accuracies of most models start to exhibit saturation at high sample sizes, with convolutional neural network models approaching near perfect classification of ten digits. **c** As a more difficult successor of MNIST, the Fashion dataset is about classifying ten categories of clothing in photos. Similar to MNIST, linear models are outperformed by kernel models, which are outperformed by deep models. In contrast to MNIST, the performances of the model classes are harder to distinguish for low sample sizes and begin to fan out with growing sample size. In the Fashion dataset, more images are necessary for kernel and deep models to effectively exploit nonlinear structure to supersede linear models. **b**, **d** Image-derived brain phenotypes (IDPs) provided by the UKBiobank were used to classify subjects into ten subject groups divided by sex and age. The number of categories is equivalent to and the feature number *p* is similar to MNIST and Fashion. In both commonly acquired structural (sMRI) and functional (fMRI) brain images, linear, kernel, and deep models are virtually indistinguishable across all examined training image sets and prediction accuracies do not visibly saturate. Moreover, using different tree-based high-capacity classifiers did not outperform our linear models as well (Supplementary Fig. 1). To the extent that complex nonlinear structure exists in these types of brain images, our results suggest that this information cannot be directly exploited based on available sample sizes. The number of input variables in a modeling scenario is denoted by *p*. IDP = image-derived phenotype. Error bars = mean ± SD across 20 cross-validation iterations (all panels).

To carefully quantify the degree to which nonlinear structure can be exploited in MNIST and Fashion datasets, we compared the performance of linear models and nonlinear kernel SVMs with gradually increasing number of training images. Systematic improvements in prediction accuracy when elevating from linear models to nonlinear models consistently indicated the existence of exploitable nonlinear information that was predictive of the digit category in the dataset. On the MNIST dataset (Fig. 2a), linear and kernel models performed indistinguishably for low sample sizes. Yet, we observed that linear models and kernel models have diverged in prediction accuracy starting at around 1000 example images. Exceeding this sample size, the worst among the three kernel models (RBF-SVM) outperformed the best among the three linear models (logistic regression). In the Fashion dataset (Fig. 2c), the worst kernel model (sigmoid-SVM) began to outperform the best linear model (logistic regression) starting from 4000 observations. This difference in performance scaling grew to 4.03 percentage points (p.p.) and 1.76 p.p. at 8000 observations for MNIST and Fashion, respectively.

That is, we show a less prominent gap between linear and shallow nonlinear methods in one of two comparable datasets with a more complicated prediction goal (i.e., detect clothing rather than handwritten digits). For sufficiently large sample sizes available for model training, kernel methods outperformed linear models in both the MNIST and Fashion datasets. Hence, kernel SVMs could readily exploit nonlinear structure in the examined datasets that is inaccessible to simpler linear models by principle.

Next, we examined more closely how the difference in the ten-class prediction problem scales with steadily growing sample sizes in the MNIST and Fashion datasets. The sample size necessary to saturate the prediction performance of a classifier relates to the complexity of patterns that can be reliably derived from the amount and dimensionality of observations available for model training. The out-of-sample prediction performance of both linear and kernel models saturated with increasing sample size in MNIST. Performance of linear models (logistic regression) improved by 14.67 p.p. from 71.64 ± 2.62% to 86.31 ± 1.06% when the data availability increased from 100 to 1000 observations, whereas the performance for shallow nonlinear models (i.e., RBF-SVM) improved by 15.60 p.p. from 72.58 ± 2.34% to 88.23 ± 1.11%. Improvements reduced to 3.50 p.p. (5.62 p.p. for RBF-SVM) from 1000 to 8000 image observations. This saturation of prediction accuracy was less pronounced when applying the same learning algorithms to images of the Fashion dataset: linear models (logistic regression) improved by 10.22 p.p. from 69.34 ± 2.95% to 79.55 ± 1.67%, from 100 to 1000 observations, but only 4.31 p.p. from 1000 to 8000 observations. Shallow nonlinear models (SVM-RBF) improved by 11.08 p.p. from 69.50 ± 2.89% to 80.58 ± 1.57%, from 100 to 1000 observations, but only by 5.78 p.p. when growing training sample size from 1000 to 8000 observations.

In sum, consistent with our expectations, nonlinear methods such as kernel SVMs progressively outperformed linear models such as logistic regression as the number of observations available for model building grew larger. In addition, the prediction performance slowly saturated when increasing the number of observations beyond 1000 example images. Both effects were more pronounced for the simpler prediction goal of digit classification (MNIST) than in the more complex clothing classification (Fashion). That is, in the more challenging prediction problem, the richer nonlinear models could learn even more as the amount of available image data grew. More complex classification tasks needed a higher number of samples to saturate the models. Our empirical scaling results confirm that these standard machine learning datasets contain nonlinear structure that our nonlinear kernel models can take always more advantage as the numbers of training observations grows.

### Performances on brain images scales similar to linear models

To assess how the performance of common linear models and nonlinear kernel models behaves on brain-imaging data, we created a range of classification scenarios using a currently largest brain-imaging dataset—the UKBB Imaging. We tried to specifically design the classification problems to be similar to key properties of MNIST and Fashion to facilitate comparison of the results between brain-imaging and machine learning reference datasets. As a classification target, we constructed a ten-class target variable based on subjects’ age and sex in analogy to the data shape in MNIST and Fashion. Our UKBB data provided structural and functional MRI simultaneously available in 9,300 subjects. We evaluated different views of the underlying brain data, corresponding to distinct and often-used forms of brain-imaging data analysis: UKBB provides high-level imaging-derived phenotypes (IDPs) of resting-state functional brain MRI (independent component analysis-based functional connectivity) and structural brain MRI (regional gray and white matter features), which brought the >100,000 gray matter voxels per brain scan to a dimensionality that is akin to the dimensionality of MNIST and Fashion: *p* = 1485 variables for functional MRI and *p* = 164 variables for structural MRI. In addition, to explore data analysis scenarios focused on voxel-level statistics (e.g., general linear model such as implemented in Statistical Parametric Mapping and other common brain-imaging analysis software packages), we reduced the raw sMRI voxels to MNISTs dimensionality of 784 variables by different feature selection and dimensionality-reduction techniques. Finally, in preparation of our analysis of modern convolutional neural networks, we extracted central two-dimensional sMRI slices for each anatomical plane.

**Fig. 3:**
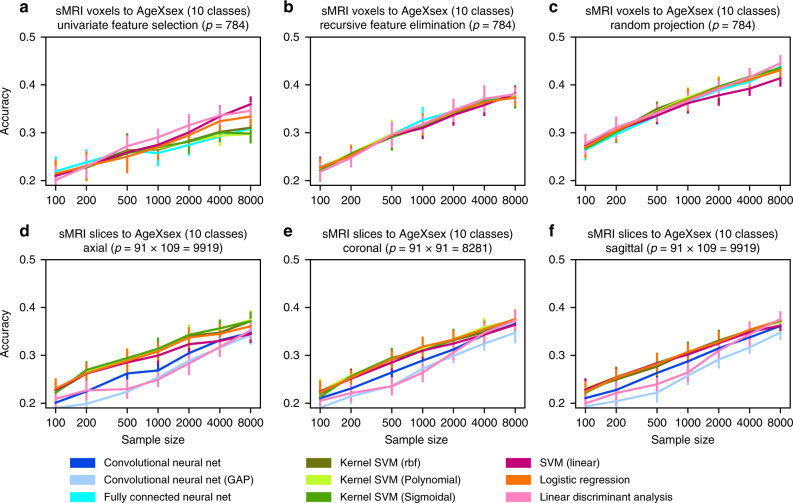
Powerful predictive algorithms fail to improve classification accuracy for several views on brain images. **a**–**c** Whole-brain gray matter information from structural brain images (sMRI, UKBiobank) was summarized by three widely used feature selection/engineering techniques. The original tens of thousands of gray matter voxel volumes were reduced to 784 variables for comparability to the dimensionality of MNIST and Fashion (Fig. 1), as a basis for learning predictive patterns for classifying ten sex/age groups. Prediction accuracies achieved using univariate feature selection (relevance tests independent for each variable) are outperformed by recursive feature elimination (accounting for conditional effects between variables), which in turn is superseded by random projections (low-rank transformations of all original variables). None of these dimensionality-reduced brain images lead to systematic differences between linear (red tones), kernel (green tones), and deep (blue tones) model classes. In particular, fully connected deep neural networks did not visibly outperform the examined classical linear or kernel methods. **d**–**f** Axial, coronal, and sagittal slices of whole-brain gray matter images (sMRI) were used for a ten-group categorization. In contrast to classifying 2D images of digits and clothing into ten categories (Fig. 2a, c), classifying 2D images of brain anatomy into ten age/sex groups does not exhibit obvious performance differences between linear, kernel, and deep models. These analyses again indicate scarcity of easily exploitable nonlinear structure in common sMRI brain scans for the present sample sizes. The number of input variables in a modeling scenario is denoted by *p*. Error bars = mean ± 1 SD across 20 cross-validation iterations (all panels).

**Fig. 4:**
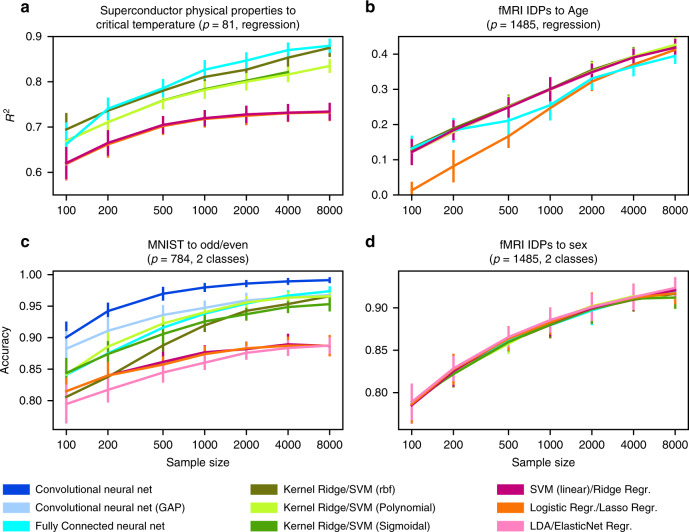
Exploitable nonlinearity is apparent in different open datasets but not in functional brain images. To complement our classification of ten age/sex-stratified groups (Figs. 2 and 3), we decoupled the stratified prediction goal by separately examining continuous age regression and categorical sex classification using linear (red tones), kernel (green tones), and deep (blue tones) models. In the superconductivity benchmark dataset (**a**), critical temperature was predicted based on 82 physical properties like thermal conductivity, atomic radius, and atomic mass^53^. Here, kernel and deep models clearly outperform linear models as measured by out-of-sample explained variance (*R*^2^, coefficient of determination). In contrast, in age prediction based on functional brain scans (**b**), the best performing linear model (Ridge regression) has scaling behavior virtually identical to kernel and deep models. This age prediction using fMRI scans is conceptually similar to previous analyses on brain maturity that reported 55% explained variance on 238 subjects aged 7–30 years^70^. These investigators noted “asymptotic maturation toward a predicted population mean maximum brain age of ~22 years […] The fitted models mainly differed in their predictions for younger ages.” In our much older UKBiobank subjects (62.00 ± 7.50 years), we reach ~40% explained variance, whereas the learning curves suggest further performance gains as more brain data become available. We identify a similar discrepancy between machine learning and brain-imaging datasets in the binary classification setting. In even (0, 2, 4,…) vs. odd (1, 3, 5,…) digit classification on MNIST (**c**), kernel and deep models diverge from linear models in classification accuracy as the sample size increases. However, the kernel and deep models are not superior in sex classification based on fMRI data (**d**), where all examined models showed virtually identical prediction performance. The number of input variables in a modeling scenario is denoted by *p*. IDP = image-derived phenotype. Error bars = mean ± SD across 20 cross-validation iterations (all panels).

**Fig. 5:**
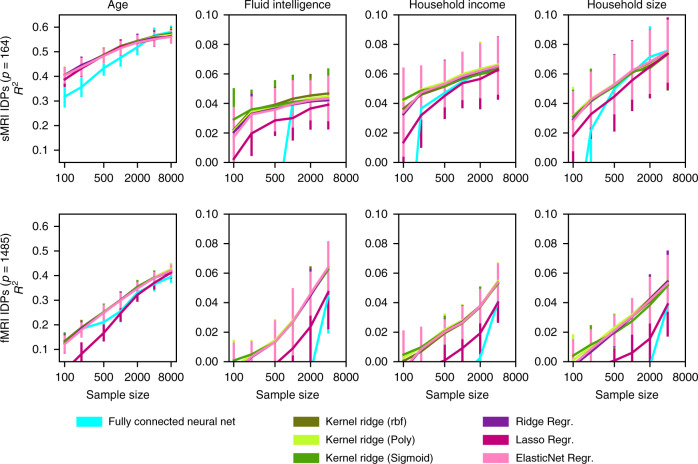
More difficult prediction goals show no evidence of exploitable nonlinear relationships in brain images. To complement the easily predictable targets of age and sex, we reiterated our benchmarking analyses on more complex demographic indices and lifestyle factors, including IQ, socioeconomic status, and household size. Ridge and ElasticNet regression provide a linear baseline that performed on par with more powerful kernel and deep neural network models in the prediction of complex target variables (*R*^2^, coefficient of determination). Lasso approached comparable performance at higher sample sizes. Moreover, the performance of linear models did not consistently saturate. Especially the sample complexity curves for predicting fluid intelligence from fMRI and household size from sMRI show that we do not appear to be close to fully exploiting the predictive information contained in brain MRI data. Please note that some prediction targets (fluid intelligence, household size, and income) were not available for all subjects, which is why the *n* = 8000 results are missing in some cases. Our observation that powerful machine learning models so far fail to beat linear baselines on brain-imaging data is not limited to the prediction of sex or age, but extends to more complex prediction targets. Error bars = mean ± 1 SD across 20 cross-validation iterations (all panels).

Although we have made an effort to tackle brain-imaging classification problems with properties similar to MNIST and Fashion in terms of number of classes, features, and sample size, we recognize that the brain-imaging classification problems are still more complex (e.g., age relates to the brain features in a continuous way and people’s brain age differently so there might be high overlap between the classes). We therefore ran additional control analyses on binary (sex) classification and continuous (age) regression (Figs. 3 and 4), as well as more complex prediction targets, such as fluid intelligence, household income, and number of people in the household (Fig. 5).

In our analyses of brain-imaging data (Figs. 2b, d, 4, and 5), we observed different scaling behavior compared to benchmark machine learning datasets for common linear models and nonlinear kernel methods. Across the different views of the brain scans, model performance converged on the same pattern of observations: for each sample size, accuracies for all examined models were mostly statistically indistinguishable. In this example of the currently largest brain-imaging dataset, we did not observe signs of accuracy saturation. That is, doubling the sample size yielded stable, mostly indistinguishable gains in accuracy. For instance, for sMRI IDPs (Fig. 2b) at a full training set of 8000 observations, the best kernel model (polynomial-SVM: 37.58 ± 2.08%) performed indistinguishably from the worst linear model (LDA: 38.26 ± 1.46%). Performance of linear models (logistic regression) grew by 5.78 p.p. from 26.02 ± 1.98% to 31.80 ± 1.92% (7.23 p.p. from 24.92 ± 1.81% to 32.14 ± 2.44% for RBF-SVM), from 100 to 1000 observations, indistinguishable from the 4.97 p.p. (5.29 p.p. for RBF-SVM) for 1000 to 8000 observations.

Qualitatively equivalent results were observed for different dimensionality-reduction techniques (Fig. 3a–c) in regression and binary classification settings (Fig. 4), and, importantly, for different prediction targets—sex, age, fluid intelligence, household income, household size, and the combined ten-class variable (Figs. 2, 4, and 5). These findings were invigorated by additional analyses that showed several high-capacity tree estimators (i.e., random forests, extremely randomized forests, and gradient boosting trees) could also not outperform our linear models based on brain images (Supplementary Fig. 1). In contrast to the examined machine learning reference datasets, brain images from the examined UKBB subjects revealed neither saturation of accuracy in our phenotype predictions with increasing sample size nor performance gains from kernel models with higher expressive capacity.

### Performance of DNN models

Finally, we compared the performance of DNN models on our brain-imaging data. Both fully connected neural network architectures and common variants of convolutional neural networks systematically differed in the performance on the machine learning benchmark datasets compared to accuracy profiles in brain-imaging data. We initially carried out a positive test to confirm that our convolutional neural network architectures, as expected^18^, successfully outperformed kernel and linear models in cancer classification from histological tissue slices (Supplementary Fig. 2).

Fully connected neural networks outperformed all linear models and all but the best performing kernel model on the MNIST and Fashion datasets (Fig. 2a, c). The same observations were not made for any examined representation of brain-imaging data. Regarding sMRI and fMRI IDPs (Fig. 2b, d), sMRI voxels (Fig. 3a–c) and whole sMRI slices (Fig. 3d–f), fully connected neural networks performed indistinguishably from both kernel and linear models. The same observation held true for our control analyses of the regression and the binary classification (Fig. 4).

Convolutional neural networks showed analogous scaling behavior. On both MNIST and Fashion datasets, convolutional neural networks outperformed the best performing kernel model by 2.93 p.p. from 95.63 ± 0.79% to 98.56 ± 0.36% and by 3.44 p.p. from 85.63 ± 1.32% to 89.07 ± 1.20%, respectively, at 8000 observations (Fig. 2a). In contrast, on sMRI brain slices, convolutional neural networks outperformed neither linear nor kernel models (Fig. 3d–f). Central axial, coronal, and sagittal slices yielded comparable prediction accuracy for logistic regression (36.09 ± 1.38%, 37.58 ± 1.81%, 37.35 ± 1.21%, respectively) and convolutional neural networks (34.74 ± 1.65%, 36.52 ± 1.83%, and 36.15 ± 1.82%, respectively). Thus, machine learning reference datasets and brain-imaging data differed in that convolutional neural networks excel on the former, but fail to improve over simpler methods on the latter.

Taken together, we described three important ways in which predictive models performed systematically differently between the machine learning reference datasets MNIST and Fashion on the one hand and brain-imaging data on the other hand. First, performance of linear models saturated with increasing sample size on MNIST and Fashion (less so kernel and deep models), but not in predicting key phenotypic differences from brain-imaging data. Second, kernel models consistently outperformed linear models in MNIST and Fashion, which was not the case in our analyses of brain-image-based prediction of interindividual demographic and lifestyle indicators. Third, deep models clearly outperformed linear and kernel models on MNIST and Fashion, but not in the analyses we have carried out here on brain images. Our quantitative investigation can therefore be taken to argue that the largest brain-imaging dataset currently at our disposal still does not reliably enable phenotype prediction by exploitation of complex configurations in data using modern pattern-learning algorithms.

### Performance of 3D convolutional DNN models

As part of the revision process, we have added a direct comparison to a state-of-the-art three-dimensional (3D) convolutional neural network^19^, which was estimated based on our full set of training sMRI scans (*n* = 8000) using our identical cross-validation splits. This neural network architecture has won the first place in the 2019 predictive analytics competition on brain-imaging data (https://www.photon-ai.com/pac2019). In our binary classification setting (male vs. female), the 3D convolutional architecture here achieved 98.93 ± 98.02/99.70% (confidence interval capturing sampling variation effects with 95% coverage) and our simple linear model achieved 98.02 ± 96.77/99.08% (L2-penalized logistic regression based on 1024 principal components). In our regression setting (age prediction), the 3D convolutional architecture here achieved *R*^2^ = 0.61 ± 0.56/0.66 (coefficient of determination) and our linear model achieved *R*^2^ = 0.61 ± 0.54/0.67 (L2-penalized linear regression based on 1024 principal components). The additional results with an established fully convolutional neural network algorithm did not change our pattern of results.

### Impact of noise on nonlinear classification

Finally, brain images measured using MRI are probably influenced by several noise sources, including thermal noise, system noise from the imaging hardware, and noise from unrelated physiological processes such as breathing, as well as other factors unrelated to brain structure or function. As such, the signal of interest from actual brain tissue represents only a part of the total measurement. In comparison to the information-dense images from MNIST and Fashion, brain images can be expected to have a substantially poorer signal-to-noise ratio.

We wondered whether a high level of noise can provide some explanations for the observed differences in sample complexity between brain-imaging and machine learning reference datasets. For this purpose, we analyzed how the sample complexity of MNIST changes with increasing contamination of the digit images with additive noise. We extracted the eight leading principal components that capture most variance in the MNIST data, as this led to the largest performance differences between linear and nonlinear models (Supplementary Fig. 3). We added separate sources of independent and identically distributed Gaussian noise to each of the latent embedding variables and used the thus noise-corrupted embedding projections as input variables into our otherwise identical classification pipeline.

**Fig. 6:**
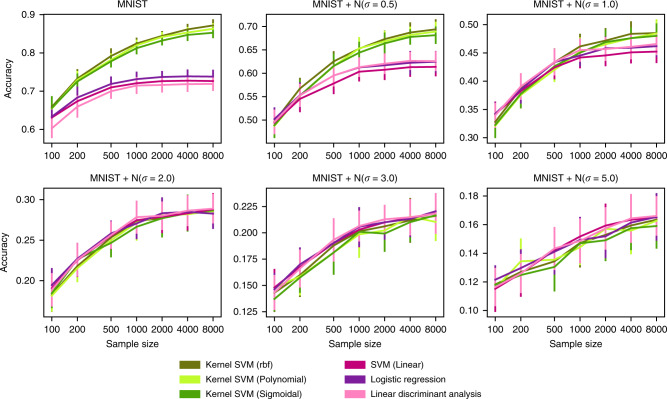
Adding noise to MNIST images leads to scaling behavior similar to that of brain images. We extracted the eight leading principal components from the digit images (MNIST) and added Gaussian noise to each principal component. The principal component embeddings were then used as input variables for the models (see Supplementary Information for details) to predict the class label (i.e., digits). Increasing the amount of noise from *σ* = 0.5 up to *σ* = 5.0 entailed two effects: first, gradual diminishing of the performance difference between nonlinear kernel models and linear models. Second, the increasing level of noise in the images reduced the extent to which prediction performance saturated with increasing sample size. At *σ* = 5.0, the sample complexity curves of MNIST data followed the same pattern as we observed on brain images (Fig. 2): kernel and linear models performed indistinguishably, and prediction performance did not visibly saturate. Our quantitative findings invite the tentative speculation that MRI brain-imaging data may be too noisy to allow capturing nonlinear relationships for phenotype prediction at currently available sample sizes. Error bars = mean ± 1 SD across 20 cross-validation iterations (all panels).

**Fig. 7:**
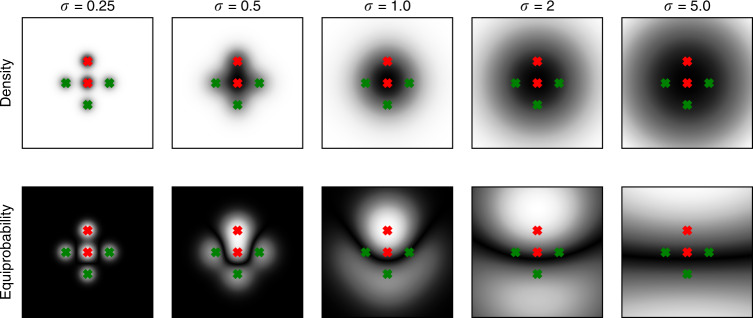
Gaussian noise can lead to linearization of decision boundaries. Shows a simple nonlinear binary classification problem consisting of two mixtures of Gaussians in 2D space with identical isotropic covariance (*σ*). Samples of the first class are generated by the two Gaussians marked with red crosses; samples of the second class are generated by the three Gaussians marked with green crosses. The first row indicates the overall probability density for the generating mixtures. The second row indicates equiprobability—i.e., *f*(**x, y**) = |*P*_red_(**x, y**) − *P*_green_(**x, y**)|—areas in which both red and green classes are equally likely (black). The resulting black margin separating the red and green mixtures represents the ideal decision boundary. Adding Gaussian noise—i.e., increasing σ—gradually turns the decision boundary from a highly nonlinear U-shape (*σ* < 2) to a linear decision boundary (*σ* > 2). Hence, in certain data scenarios, high levels of Gaussian noise can linearize an irregular decision boundary.

Increasing the amount of injected noise from *σ* = 0.5 up to *σ* = 5.0 showed two key effects (Figs. 6 and 7): First, intentionally adding random noise led to gradual narrowing of the gap between nonlinear kernel models and linear models. Second, the increasing level of noise reduced the extent to which prediction performance saturated with growing sample size. At *σ* = 5.0, the sample complexity curves (with otherwise identical analysis workflows) of MNIST data yielded a prediction performance curve that appeared reminiscent of the scaling curves from brain-imaging data, in both these aspects (Fig. 2): kernel and linear models performed indistinguishably well and prediction performance did not visibly saturate. These observations were also made when adding noise to brain images when omitting or carrying out preliminary Gaussian smoothing of the brain data (Supplementary Fig. 4). These quantitative findings would be compatible with the view that MRI brain images carry too much noise to exploit nonlinear relationships for powerful phenotype predictions using learning algorithms at currently available sample sizes.

## Discussion

Do today’s kernel methods or DNN algorithms provide advantages for phenotype predictions from brain images by drawing on shallow-nonlinear or deep-nonlinear information? Can the triumphs of kernel and deep methods in datasets from the machine learning community be mimicked in currently available brain-imaging population datasets?

Our study initiates principled answers to these increasingly important questions. We have profiled the degree to which complex nonlinear relationships can be extracted and exploited to improve phenotype prediction accuracy over linear models in structural and functional brain images from the UKBB. Our careful analyses have delineated the scaling behavior of prediction performance with increasing number of training observations for three key classes of learning algorithms: linear, kernel, and deep models. Importantly, we initially replicated performance gains escalating to nonlinear and then to hierarchical nonlinear models, as reported in performance benchmarks from machine learning. Such improvements in phenotype prediction by taking advantage of increasing model complexity were not consistently apparent in the currently largest population brain-imaging cohort.

A central finding from our analyses pertains to the performance trajectory of linear models with gradually growing sample size (i.e., empirical sample complexity). Our prediction outcomes from brain-imaging data and reference datasets from machine learning differed in the way that the prediction accuracy increased with the availability of data from additional observations. In the machine learning datasets, we expected and indeed observed saturation of prediction performance of linear models. This effect was most prominent when carrying out digit classification on MNIST by means of logistic regression and LDA: the prediction performance increased rapidly as we grew the sample size for model building from 100 to 1000 available images. Subsequently, the prediction accuracy approached a plateau. We noticed hardly any additional improvement in prediction accuracy when doubling the sample size from 4000 to 8000 example images.

In contrast, in our analyses of brain scans, this step up in the number of images available for model building yielded continuous gains in prediction performance for all examined brain-imaging modalities and data representations. Across different MRI protocols to measure brain tissue, we determined that increasing the number of subjects from 4000 onward entailed steady performance gains in prediction accuracy. Importantly, the size of the largest currently available brain-imaging repositories was insufficient to saturate the learning capacity of even simple linear models. These linear models were here applied in a multivariate fashion pooling brain information from many parts of the brain for phenotype prediction^20,21^. Our findings suggest that, approaching brain scans obtained from up to 10,000 subjects, the prediction capacity of common linear models is not yet fully exhausted.

We deem the uncovered prediction reserve for the linear modeling regime important in several ways. This scaling behavior provides new arguments for the common criticism that there may be limited information contained in brain-imaging data like MRI that can be usefully exploited for prediction in real-world applications^21^. For instance, in the case of fMRI, blood flow and oxygenation do not provide an immediate read out of neuronal activity, and operate at coarser-grained temporal and spatial scales than actual electrochemical information processing in neuron populations. Some authors have claimed that “fMRI is as distant as the galvanic skin response or pulse rate from cognitive processes^22^.” Despite these caveats, there have been a number of encouraging findings. For instance, functional brain connectivity could be shown to provide a neural fingerprint to make accurate predictions of interindividual differences in cognitive performance^23^. Yet, such promising reports are controversially discussed in the neuroscience community and have been flagged as hard to replicate by some investigators^24^.

Our results favor the more optimistic interpretation scenario. We suggest that neuroscientists are not yet fully exploiting predictively useful information in brain-imaging data. Not even simple linear models have reached plateaus of performance in our predictions of sex, age, and other phenotypes from common MRI measurements at present sample sizes. As such, we are likely to be far from reaching the limits of single-subject prediction accuracy by leveraging brain-imaging data.

Our finding of unexhausted linear modeling reserve may have considerable ramifications. This is because systematic evaluations of modern machine learning in brain imaging^13,25,26^ and a large amount of studies applying complex nonlinear models in brain imaging often have operated under the implicit assumption that linear effects are already sufficiently characterized with their prediction scaling as sample size increases. Typically, carefully characterizing linear effects provides a solid basis to compare against more complex nonlinear models. Many elaborate machine learning methods can be viewed as extensions of classical linear regression. If there is insufficient data to estimate the parameters of a simple linear model, then it is even less likely that the parameters of even more data-hungry nonlinear models can be estimated with satisfaction. The recommendation emerging from the present investigation is that regularized linear predictive models are likely to serve as a formidable starting point^27^ for single-subject prediction in larger future brain-imaging datasets in health, and potentially also disease, for the foreseeable future.

If nonlinear intervariable relationships exist in a given dataset and are exploitable for a specific prediction goal and available sample size, we expect a step up in prediction performance as we upgrade classical linear models to always more expressive models. In a set of analyses, we confirmed the expected increase in prediction performance with growing capacity to represent complicated patterns in one of the most widely endorsed machine learning benchmark datasets (digit image classification in MNIST), as well as a more difficult companion dataset (clothing image classification in Fashion). In particular, kernel methods consistently outperformed commonly used linear models, with accuracy gains of 4.03 and 1.76 p.p. on MNIST and Fashion, respectively, on average.

Our ability to quantify the detection of nonlinear intervariable configurations in MNIST and Fashion to distinguish ten categories of images (ten digits or types of clothing) corroborates our application of kernel SVMs as a viable and effective tool to test for the existence of predictive nonlinear patterns in data from the brain-imaging domain. If information carried in these brain measurements are related to the prediction goal in complicated ways, an SVM with a nonlinear kernel extension is expected to reliably outperform linear models when provided with brain scans from enough subjects.

However, we did not observe systematic performance gains in prediction accuracy when we examined brain images from UKBB, although our study empirically replicated them in our analyses on the MNIST and Fashion datasets. We found that none of the three nonlinear kernel models clearly outperformed linear models on structural or functional brain scans. This finding is particularly apparent in the fMRI data widely used for computing functional connectivity between brain regions and networks, where all examined models perform nearly identically across the sample sizes empirically simulated in our study. In fact, kernel and linear methods performed virtually indistinguishably in a wide range of phenotype prediction analyses of brain-imaging data from the currently largest biomedical dataset—the UKBB—designed to be approximately representative of the UK general population. A similar conclusion on fMRI data has been drawn years ago by Cox et al.^28^. These investigators noted that “in spite of many conceivable sources of nonlinearity in neural signals, the nonlinear […] SVMs used did not significantly outperform their linear counterparts.”

Limited gains in phenotype prediction performance from adopting always more elaborate nonlinear models may turn out to be a common property of several brain-imaging analysis settings with sample sizes in the order of thousands of subjects. We therefore examined different neurobiological measurements as obtained by 3T MRI scanning lending insight into brain anatomy and intrinsic functional coupling between brain regions and large-scale brain networks. We further assessed different representational windows into the brain scans, from the application of various data representation methods to handcrafted features to raw (one-dimensional) voxel data to feature engineering/selection applications to whole-brain features. Moreover, we have considered different prediction goals—such as  age, sex, and stratified age/sex groups—known to explain large amounts of variability in brain MRI data^4^. Nevertheless, in our analyses of canonical machine learning datasets, but not in brain-imaging data, linear models were reliably outperformed by all examined nonlinear kernels. Even easy-to-predict phenotypes, such as age and sex, failed to show reliably improved prediction performance when escalating to current nonlinear models for sample sizes in the order of thousands of subjects. Thus, we are enticed to speculate that even harder to define and trickier to measure concepts, such as IQ, social cognition capacity, and mental health diagnoses, may seldom achieve performance gains from deploying more complex models at similar sample sizes.

Our benchmark study thus provides new quantitative evidence bearing on an increasingly important question: how well can demographic and lifestyle factors be derived from commonly acquired brain scans? The fact that most analyses showed highly comparable prediction accuracies between linear models and models with kernel extensions in brain images allows for several possible interpretations. On the one hand, it may be the case that there exist few salient nonlinear relationships in the examined types of brain data that are useful for forecasting interindividual differences in phenotypes. In this case, linear models would be expected to be more data-efficient and effective at extracting the crucial patterns that are instrumental for the prediction goal. On the other hand, our noise-inducing empirical simulations speak more to the possibility that nonlinear configurations may truly exist in our brain-imaging data, but cannot be easily used to serve the examined phenotype prediction goals given the size of currently available brain-imaging repositories. The nonlinear interactions in brain-imaging data could be so intricate or noisy that it would take a substantially larger sample size to reliably capture and leverage them in practice. In addition, the lens through which the here charted nonlinear methods “see” general patterns in the data may not be aligned well with the kind of nonlinearity present in these types of brain scans (i.e., mismatch of inductive bias). As a limitation of the present investigation, we cannot provide a definitive answer as to which of these possibilities is more pertinent.

As a practical consequence, the scarcity of exploitable nonlinear structure in our brain-imaging data suggests that linear models will continue to play a central role as bread-and-butter approach to analyzing brain scans like MRI measurements, at least over the next years^2,8,13^. The additional representational expressivity that modern nonlinear models provide comes at the cost of a more serious risk of overfitting^29^ and typically added challenges in interpretability of the modeling solutions^8,30–32^. Our empirical results suggest that, for sample sizes available today, the added costs of implementing one of the current more complex models in computational requirements and technical knowledge may rarely be justified by the theoretical potential of achieving better prediction accuracy on regular MRI brain scans.

Finally, we contemplate our findings regarding the value of contemporary DNN algorithms for the imaging neuroscience community. Three trends appear to stand out in the existing brain-imaging literature. First, it is noteworthy that there is still a suspicious scarcity of published MRI or positron emission tomography studies that unambiguously demonstrate substantial gains from applying deep learning techniques. In line with this, Vieira and colleagues^25^ noted that “despite the success of [deep learning] in several scientific areas, the superiority of this analytical approach in neuroimaging is yet to be demonstrated.” In domains such as computer vision or natural language processing, DNNs have already dramatically improved state-of-the-art prediction performances^11,33^. However, in the application of neuroimaging data analysis, a similar revolution has not materialized for most common prediction goals, despite investment of considerable research efforts. However, a few successful exceptions have shown advantages of deep learning applied to neuroimaging data over conventional approaches, such as for the specific goals of image segmentation^18,34–37^ and image registration^38,39^.

Second, deep learning approaches have repeatedly been found to perform worse or indistinguishably well compared to simpler baseline models when predicting demographic or behavioral phenotypes^13,40^. For instance, Cole et al.^40^ showed that deep convolutional neural networks did not outperform Gaussian process models when predicting brain age from sMRI brain scans in ~2000 healthy subjects. Consistently, He et al.^13^ found that three different DNN architectures did not outperform kernel regression models at predicting a variety of phenotypes, including age, fluid intelligence, and pairs-matching performance from whole-brain connectivity profiles derived from fMRI  brain scans. Moreover, in the 2019 ABCD challenge, kernel ridge regression outperformed deep learning approaches in predicting fluid intelligence from sMRI data^41^. The four best predictive modeling approaches did also not utilize deep learning in the TADPOLE challenges to predict progression in Alzheimer’s disease^42^. In general, even many reports of modest improvements from using DNNs are controversially discussed in the brain-imaging community.

Third, contrary to many scientists’ expectations, gains from machine learning algorithms with the ability to represent complicated nonlinear relationships in brain-imaging appear to decrease or stagnate when incorporating data from more sources or acquisition sites. This recurring observation suggests overfitting or possible publication bias. The general expectation is that improving model building with additional training observations should improve prediction performance of complex nonlinear models, especially when moving from dozens or few hundreds to thousands of subjects. In contradiction with this plausible intuition, Arbabshirani et al.^43^ pointed out that “the reported overall accuracy decreases with sample size in most disorders.” Specifically, in the context of deep learning, Vieira et al.^25^ noted that “the pattern of difference in performance did not seem to vary systematically with sample size.” Woo et al.^21^ concluded that their “survey reveals evidence for such [publication] bias in predictive mapping studies.” These circumstances were speculated to reflect increasing heterogeneity of larger patient samples and inter-site differences in the process of data acquisition. However, these considerations also cast some healthy doubt on the high expectations about embracing DNN applications to the types and amount of brain-imaging data that exist today.

Our findings from comprehensive model profiling dovetail with these earlier reports and observations from previous imaging neuroscience studies. Certain DNN models consistently outperformed all kernel models and all linear models in our analyses of MNIST and Fashion datasets. We did not witness similar effects in our analyses of brain-imaging data. Here, the majority of models performed statistically indistinguishably for several investigated imaging modalities, data representations, and predicted target phenotypes. This lack of consistency in performance differences between model classes—in the world’s currently largest biomedical dataset—is instructive. The present null result lends credence to reports of the potential for overfitting or publication bias in the field of brain imaging.

In carefully controlled experiments, even null results can be evidence of absence. However, due to the high flexibility of deep learning, it is nearly impossible to fully explore all possible combinations of hyperparameters and model architecture choices^11^. Hyperparameter search is computationally expensive. As such, only a well-chosen subset of candidate hyperparameter combinations can reasonably be evaluated in any given empirical study. Thus, negative results in training DNNs are often disregarded as these outcomes leave open the possibility of insufficient hyperparameter tuning. A common response to a negative result in deep learning is to challenge the range or granularity of the hyperparameter grid or to question the model architecture or the data preprocessing choices. We are aware that the same objections could be raised with regard to our own results. However, the lack of exploitable nonlinearity for phenotype prediction also holds based on our linear and kernel model results on MRI brain scans alone, disregarding our deep learning applications altogether.

Previous work from the neuroimaging community^13^ has made similar claims in stating that DNNs did not successfully outperform kernel regression models in the case of behavior prediction from functional connectivity. Yet, their experimental analysis setup may not be able to fully dismiss the critique of insufficient hyperparameter optimization in deep models. In this way, our work provides a critical addition to the existing literature, by lending some support to the idea that kernel models for exploiting nonlinearity might not even be expected to outperform simpler linear models^3,30^. As such, hurdles in exploiting even more sophisticated hierarchical nonlinearity may have been anticipated before the deep learning era in brain imaging.

Although still an active area of research, two theoretical attempts are generally evoked to explain the exceptional performance of DNNs on a variety of applications; besides the mathematical proofs that deep models are able to approximate arbitrarily complex prediction rules given sufficient training observations (i.e., universal approximation theorem). One view states that the hierarchical, compositional nature of deep learning allows for a particularly parsimonious representation of some forms of nonlinear structure embedded in data^44,45^. In analogy to a world map, the globe can be seen as compositional of continents, which split into countries, which split into provinces, which split into cities, and so forth. This hierarchical structure may be efficiently represented by multiple nonlinear processing layers in artificial neural network models, where individual layers correspond to streets, districts, cities, and so forth (but see ref. ^46^). The same nested structure applies to human language and thus written text and recorded speech data.

Based on this conceptualization, one should expect DNNs to improve upon linear and kernel models in a given application domain only if there exists exploitable hierarchical and nonlinear information in the data. Compared to kernel methods, DNNs tend to have orders of magnitude more parameters and often require a correspondingly high number of training observations. Many recent empirical successes of deep learning are largely attributed to the growing availability of extremely large datasets, especially in domains building on Internet-scale data of images and natural language, as well as the availability and affordability of computation^11^. DNNs appear to violate theorems of statistical learning theory in that some highly overparameterized models still generalize unusually well to new observations^47^. Nevertheless, we generally expect kernel methods to require fewer training observations to extract useful nonlinear structure in data^48^. In addition, kernel methods typically have only few hyperparameters. A smaller number of complexity parameters allows for a more comprehensive search of the optimal model subspace for the data at hand. Given their possibly more efficient empirical hyperparameter tuning, kernel methods thus have the practical potential to select model instances that are better suited to identify and exploit nonlinear interactions existing in the data in smaller sample sizes than DNNs. However, common kernel methods do not have the ability to take advantage of complex hierarchies in representing patterns in the data effectively. Thus, kernel methods are, arguably, preferable when the data do not contain exploitable hierarchical structure^13^.

The second commonly evoked explanation for why DNNs may outperform earlier quantitative methods is specific to convolutional DNNs. This type of network was purpose-designed specifically for processing natural images. Convolutional neural networks were loosely motivated by the organization of the visual cortex, in that individual neurons respond to stimuli in only a limited receptive field. These biologically inspired models exploit the locality of information in images—the way in which neighboring image pixels describe the same phenomenon. In rough analogy to the visual cortex, convolutional neural networks consist of a hierarchy of layers of locally sensitive feature detectors. In contrast to visual cortex biology, each feature detector is recycled—convolved—for each position in the image. This reuse of feature detectors at different positions leads to the inbuilt bias called translation invariance. For example, a feature detector sensitive to a cat would work independently of where the cat is located in an image. This inductive bias for translation invariance (cf. introduction) introduces a useful and domain-compatible simplification in the analysis of natural images. This is because translation invariance is an important property of the physical world—a cat remains a cat independently of where in space the animal is located.

However, in contrast to MNIST, Fashion, and most other common computer vision datasets, the brain has a naturally meaningful topography as captured by an MRI image. The scanned individualsʼ head position in the scanner and the subsequent mapping to standard atlas space is widely established. For a majority of the possible prediction goals, there is no need for a translation invariant feature detector to screen the whole volume for a particular representational phenomenon—any given brain region is located in a predefined, biologically meaningful space. Some authors may therefore deem it unlikely that convolutional neural networks can invariably improve performance in phenotype prediction based on standard-resolution brain images in common reference space.

Importantly, the take-home message from our present work is *not* that DNNs will never be a useful tool for the brain-imaging field. For instance, we anticipate, deep convolutional neural networks should become particularly useful when modeling anomalies that can be anywhere in the brain, such as for detecting brain tumors or quantifying abnormal white matter lesions in multiple sclerosis^35,49^. Deep convolutional neural networks should also be valuable in servicing the neuroscience community by segmenting brain data into biologically meaningful territories, such as outlining the anatomy of cortical laminae in ultra-high-resolution brain scans^37^. As another possible avenue towards more powerful brain-behavior predictions, new neuroimaging repositories could target much fewer subjects than the UKBB with many more brain measurements for each subject in a precision mapping approach^50–52^. Nevertheless, our collective results caution that for a variety of brain-imaging applications, there may be little hard advantage to using the latest convolutional DNN models for answering neuroscientific research questions at today’s sample sizes.

## Methods

### Three reference datasets

The MNIST dataset^15^ may be the most used reference dataset for research and development in the machine learning community. Its properties are well understood because a large number of models have been benchmarked on MNIST. This classical dataset provided a convenient starting point for the present study, allowing us to reproduce and quantitatively characterize different properties of machine learning algorithms in a controlled setting. MNIST provides 70,000 images of handwritten digits (“0”–“9”) to be classified based on the raw pixel information. Each of these grayscale images consists of 28 × 28 pixels, i.e., 784 intensity values in total per digit image.

To quantitatively characterize the effects of more complex data patterns with a more challenging prediction goal, we also profiled our predictive models on Zalando’s recent Fashion dataset (“Fashion-MNIST”^16^). Instead of handwriting, the dataset consists of grayscale images of fashion products. Images are to be classified into ten types of clothing (e.g., t-shirt, sweater, and dress). MNIST has sometimes been found to be too easy to predict for very recent machine learning methods. The Fashion dataset was created with the intention to provide a more difficult pattern recognition problem than MNIST, while preserving the same number of classes (10 clothing categories), feature dimensionality (784 pixel intensities), and sample size (70,000 images). This setup conveniently allowed for using the same model architectures on both machine learning benchmark datasets and facilitated comparisons of model performance scaling.

Our aim was to compare the known properties of MNIST and Fashion datasets to common types of brain images acquired in humans (rather than an exhaustive benchmarking of deep learning in neuroimaging in general). Such direct juxtaposition allowed identifying settings where model behavior extends from machine learning datasets to brain-imaging datasets. In addition, these analyses allow developing some first intuitions of the settings where extrapolation of effects should not be expected. UKBB imaging was a natural choice for the motivation behind our experiments. This resource is the largest existing biomedical dataset to date. Our data request of the UKBB brain-imaging initiative provided structural and fMRI data for ~10,000 subjects from the same scanning site (see this illustration to get a sense of the scale of the brain-imaging challenges: https://www.youtube.com/watch?v=DbPNscjIC6U, UKBB application number 25163, information on the consent procedure can be found at biobank.ctsu.ox.ac.uk/crystal/field.cgi?id=200). We centered our analysis on a complete set of UKBB individuals who simultaneously provided the data from both imaging modalities of brain structure (sMRI) and function (fMRI), which resulted in a total sample size of 9300 data points with brain images.

From the UKBB release, we compiled a multifaceted set of brain-imaging data, representing different modalities and different views on a given modality. We derived four working datasets based on the brain images as follows: (a) intrinsic neural activity fluctuations measured by fMRI data, with pre-computed independent component analysis yielding 100 spatiotemporally coherent neural activity patterns, resulting in a feature space of 1485 connectivity strengths between cleaned “network” components estimated using partial correlation analysis. (b) One hundred and sixty-four atlas-derived features describing structural (T1-weighted MRI) MRI gray and white matter regions, as well as fiber tract summary statistics. For both region volume and fiber bundle microstructure estimates, the biologically meaningful features were provided directly by UKBB as image-derived phenotypes^4^. (c) Approximately 70,000 raw T1 voxel intensities, after gray matter masking. (d) Finally, axial, sagittal, and coronal T1 slices at the origin with resolutions of 91 × 109, 91 × 91, and 91 × 109 voxels, respectively. Further details on data acquisition, data preprocessing, and IDPs are provided elsewhere^4^.

For the sake of comparability with the MNIST and Fashion datasets from machine learning, we generated a prediction target of 10 classes from all possible combinations of two sexes and five age quintiles. That is, the UKBB subjects were divided into five male subgroups with four age cut-offs between 40 and 70 years, and five female subgroups with these four age cut-offs. As our brain-imaging dataset provided a total of 9300 individuals with multi-modal imaging information, at the time of our analysis, we randomly subsampled the much larger MNIST and Fashion datasets to the same sample size in our analyses.

In addition, we used the subjectsʼ age at scanning time and the UKBB variables fluid intelligence (field 20016), number of people in household (field 709), and household income (field 738) as prediction targets for regression analyses. Although we have designed brain-imaging classification problems with a similar number of classes, features, and example observations as the MNIST and Fashion datasets, the brain-imaging classification tasks were still expected to be much more complex than the classification problems posed in MNIST and Fashion.

Our main reference datasets, MNIST and Fashion, provide classification tasks. To show that our results and conclusions transfer to the regression setting, we additionally used the “superconductivity” dataset^53^ as a regression benchmark dataset (i.e., with a continuous outcome prediction goal). Here, critical temperature needs to be predicted as a function of 82 physical properties such as thermal conductivity, atomic radius, and atomic mass.

### Preprocessing procedures

In all analysis scenarios, the input variables were appropriately standardized by scaling variance to one and centering to zero mean across observations. In the special case of convolutional DNN models (cf. below), the images were standardized on the aggregate pixel statistics to keep all pixels on the same scale^54^.

For sMRI images, we applied an additional dimensionality-reduction step for the purpose of comparability with MNIST and Fashion. To keep the model complexity comparable between datasets (related to the input variables and thus the number of model parameters to be fitted when building a prediction model), sMRI data were reduced to 784 features, such as MNIST/Fashion, by three dimensionality-reduction methods. Each of these feature screening approaches followed a popular, but distinct approach to perform feature selection/engineering as follows^11,55^: (a) univariate feature selection (F-tests) selected input variables under the assumption of statistical independence between variables. (b) Recursive feature elimination was used in four steps of logistic regression, each discarding the 25% least predictive input variables of the current active variable set in each step^56^. This dimensionality-reduction strategy selects features under joint consideration of the current set of input variables. (c) Gaussian random projections were used to re-express the original input variables in a set of latent factors that rely on underlying low-rank structure across the whole set of input variables^57^.

### Linear models

Conceptually, three classes of models—linear, kernel, and deep—were used to comprehensively evaluate the prediction performance on each dataset (i.e., MNIST, Fashion, and UKBB). Each level of model complexity represents the state of the art of a key epoch in data analysis^9^: regularized linear models to handle large numbers of input variables (~1990–2000), kernel SVMs became go-to methods for many applications in bioinformatics and beyond from late 1990s (~2000–2010), and DNN algorithms in turn became an exponential technology very recently (~2010–2020). Within each of these distinct model classes, we used three commonly employed representative models, attempting to cover the range of approaches within each particular modeling class.

For the class of linear methods, we have selected LDA, logistic regression, and linear SVMs (without kernel extensions). LDA is a popular generative classifier that finds linear combinations of features that best serve to separate classes among the observations, which is closely related to other covariance-based analysis methods like PCA^58^. L2-regularized logistic regression^59^ and linear SVMs^60^ are commonly used discriminative models, and represent frequently chosen instances of generalized linear models and linear maximum-margin classifiers, respectively. Penalty terms for regularization constraints were tuned by grid search.

### Nonlinear models

In practice, many classification problems are not linearly separable in the original variable space. The classes to be distinguished can become separable after nonlinear transformation of the input data into a representationally richer high-dimensional space. Solving a prediction problem in this high-dimensional representation tends to be computationally prohibitive and became more tractable in practice by means of the kernel methods.

Kernel methods^10^ are able to efficiently map to high-dimensional input spaces by never explicitly computing coordinates of all data points in this enriched space, but relying on the pairwise similarities between observations instead. Many linear methods can be extended to the nonlinear regime by applying this so-called kernel trick. Arguably, the most popular kernelized estimators are kernel SVM variants^10^. These feature embedding extensions are still pervasively used today in many application domains. Key reasons include their well-understood theoretical properties, robust estimation, and often competitive real-world performance. The class of kernel approaches was, and often remains, the go-to choice to identify and use complex nonlinear interactions to the extent that these exist in the data.

We therefore decided to evaluate kernel SVMs with three of the most commonly used kernel types as follows: (a) the RBF kernel, which maps the data to bell curves centered around observations, and is popular partly because of its universal approximation capacity^61^. (b) The polynomial kernel, which maps to polynomial expansions of the original variables, which made these variable enrichments popular in text processing using natural language processing as this input expansion explicitly takes into account combinations of features^62^. (c) The sigmoidal kernel, which maps to the hyperbolic tangent—analogous to the activation function that introduce nonlinearity in the units of DNN architectures^11^—and is popular due to its relation to the shallow “perceptron” artificial neural networks^63,64^. These three instances of kernelization for nonlinear enrichment of input variable information are probably the most frequently used and the ones implemented in the dominant software packages (e.g., scikit-learn).

### Hierarchically nonlinear models

In an increasing number of application domains, single nonlinear enlargement of the input variables have been superseded by DNN models. As an extension of linear and kernel methods, DNNs can extract and represent even more complex patterns in data using an automatically derived hierarchy of nonlinear operations on the set of input variables^11^. This nested design principle allows the learning architectures to pick up on progressively more abstract intermediate representations from the data themselves—a form of automatic feature engineering that was static in kernel methods^11^. Even though more exotic artificial neural network architectures exist, fully connected DNNs and the more recent convolutional DNNs are among the most often employed types.

Analogous to our analyses based on linear and kernel models, we evaluated several common DNN architectures as follows:

(a) Fully connected neural network algorithms: the input layer has *p* units for *p*-dimensional input data. The input layer was followed by two fully connected hidden layers of 800 units each, with rectified linear unit (ReLU) nonlinearities and 50% dropout probability. These consecutive nonlinear operations were followed by a final fully connected layer of 10 units, followed by a softmax output function corresponding to predicting the probability of the target classes to be discriminated.

(b) For the small (*p* = 28 × 28) MNIST and Fashion images, convolutional DNNs consisting of two sets of convolutional and max-pooling layers (with 16 and 32 filters of 3 × 3 pixels respectively, a 2 pixel pooling size, and ReLU nonlinearities), followed by a fully connected layer of 128 units with ReLU nonlinearities, and a final fully connected layer of 10 units with softmax output corresponding to the target classes. Most tutorials and examples (e.g., for TensorFlow and PyTorch) use variations of these architectural building blocks.

(c) For completeness, we implemented a third architecture in which the final max-pooling operation is replaced by GAP—a popular approach to avoid overfitting by reducing the total number of model parameters^65,66^.

For the larger (*p* = ~100 × ~100) brain-image slices, we inserted two extra convolutional and max-pooling layers to achieve a sufficient reduction in dimensionality before connecting to the prediction-generating output layer.

The parameters of the deep models were trained with the ADAM optimization algorithm^67^: 160 epochs, with 500 gradient updates per epoch, a batch size of 32, learning rate reduction by 0.5 after 3 epochs without improvement, early stopping after 10 epochs without improvement. All deep models were trained with training-set-dependent levels of L2 regularization as the only hyperparameter.

As part of the revision process, we also performed a comparison to a state-of-the-art 3D convolutional neural network^19^, which was trained on our full set of sMRI scans (*n* = 8000) using the identical cross-validation splits. We used Peng’s computer code to apply their algorithm architecture on the here examined sMRI images. More details of Peng’s model can be found in their paper. Briefly, this model architecture was motivated by VGGNet, but reduced to ~3 million model parameters. Among seven model blocks, five blocks included 3 × 3 × 3 three-dimensional convolutional layers, batch normalization, max pooling, and ReLU activation layers. Block 6 included a 1 × 1 × 1 3D convolutional, batch normalization, and ReLU activation layers. Block 7 included average pooling, 50% training dropout, fully connected layers, and softmax output layers. The unabridged technical details and full implementation of re-using this 3D convolutional neural network for our brain images are open to any reader at https://github.com/maschulz/deeperbrain/tree/master/subanalyses/3d.

It is important to note that the purpose of our study was not to benchmark highly specialized DNN architectures from the recent neuroimaging literature. Instead, we decided to rely on established best practices to implement simple, straightforward architectures as representatives for commonly used deep learning approaches.

### Model selection and model evaluation

To estimate the prediction accuracy that we expect to obtain in new observations sampled from the population, cross-validation was computed as a gold-standard to obtain out-of-sample accuracies^55^. We have repeatedly split the observations, sometimes called Monte-Carlo cross-validation, into a training set, as well as a validation set used for model selection (i.e., hyperparameter choice) and a test set used for model evaluation with 650 observations each in the sample complexity analyses (cf. below). In the UKBB, we considered 9300 subjects with the brain images of interest. This random splitting was repeated 20 times in each modeling scenario. Training, validation, and test set were drawn exactly once per training sample size and per splitting iteration, such that the different models operated on the exact same data splits.

To carry out model selection, hyperparameters were handled in a data-dependent way by grid search^11^, separately for each dataset, model, training set size, and splitting iteration. Hyperparameter grids were set up for each model based on best practices from the literature, and chosen empirically based on relative prediction accuracy on the validation set. LDA has no hyperparameters to tune. For logistic regression and SVMs, the regularization parameter C and gamma were distanced in powers of two. Regarding hyperparameter grids for kernel models, coefficients of polynomial and sigmoidal kernels were −1, 0, or 1, and the degree of the polynomial kernel was set to 2. After the best hyperparameter combination in the grid of candidate choices had been determined, the actual absolute accuracy was assessed on the unseen test set.

Although our linear and kernel models completed hyperparameter selection and parameter estimation in the order of minutes, DNNs took orders of magnitude longer. For computational feasibility, DNNs were tuned only with regard to the L2 penalty on their parameters (0.0, 1e − 3, 1e − 5, 1e − 7 for convolutional DNNs and 1e − 4, 1e − 3.75,…, 1e − 0.5 for fully connected DNNs). Every architectural choice, such as whether or not to use dropout, filter or unit numbers per layer, learning rates, can be viewed as a form of hyperparameter choice for the model and could potentially be fine-tuned. A focus on tuning the regularization strength is most often used as a middle ground between completeness and computational feasibility in the literature^11^. Please note that all candidate sets of hyperparameter choices were evaluated exclusively on validation set data. In addition, all multi-class analyses were carried out as one-vs.-rest schemes for comparability^55^. In contrast to other authors^68^, our investigation was not focused on benchmarking multi-task learning in MRI data. Several classical and successful machine learning algorithms (e.g., SVMs) are restricted to binary classification by construction, which frustrates attempts to cleanly separate potential prediction performance gains from exploitable nonlinearity and gains from multi-task learning. Moreover, our model tuning scheme was consistent with a continuous Bayesian alternative to hyperparameter search (Supplementary Fig. 5) and robust to more detailed diagnostic tests (Supplementary Fig. 6).

### Sample complexity analysis

To satisfy the core motivation behind the present investigation, we quantified how a given model’s prediction success scales as a function of the sample size at current disposal. For each model and dataset, we ran separate cross-validated prediction analyses for increasing steps of training set sizes: *n* = 100, 200, 500, 1000, 2000, 4,000, and 8000. The resulting prediction estimates provided the basis to build a so-called learning curve^11,69^. We will call the resulting relation between the available number of observations and prediction performance the empirical sample complexity of a given model on a particular dataset and a specific prediction goal.

Such learning curves of pattern-recognition algorithms typically follow an inverse-power law. The accuracy often increases rapidly in the beginning and then slowly saturates^11^. These diagnostic assessments are typically further characterized by a saturation point and a saturation velocity. The saturation point provides a sense of the maximal performance that the prediction algorithm can achieve as the sample size keeps growing infinite to always more observations. The delineation of a model’s scaling of predictive performance is directly tied to the signal-to-noise ratio of the given dataset and the expressive capacity of the given model^11^. The saturation velocity indicates how many observations are necessary to approach maximal prediction performance and is further tied to the complexity of the target function to be approximated by the model in the dataset. Different scaling behavior of linear, kernel, and deep models can delineate the extent to which exploitable nonlinear structure is present in the data, and at what sample sizes such nonlinear information becomes practically relevant for realizing better phenotype predictions.

### Reporting summary

Further information on research design is available in the Nature Research Reporting Summary linked to this article.

## Supplementary information

Supplementary Information

Reporting Summary

## Data Availability

MNIST and Fashion data are freely available at http://yann.lecun.com/exdb/mnist/ and https://github.com/zalandoresearch/fashion-mnist. The brain data used in this work was obtained from UKBiobank under Data Access Application 25163 and (as with all UKBiobank data) are available to any bona fide researcher upon data access application to UKBiobank (http://www.ukbiobank.ac.uk/register-apply/).

## Code availability

Source code is available at http://github.com/maschulz/deeperbrain.
