# Supplementary material for: Different scaling of linear models and deep learning in UKBiobank brain images versus machine-learning datasets
Source: Nat Commun. 2020 Aug 25;11:4238. doi: 10.1038/s41467-020-18037-z (PMC7447816; doi:10.1038/s41467-020-18037-z)
Supplement: Supplementary file 1 — Supplementary Information [file 41467_2020_18037_MOESM1_ESM.pdf]

**Supplementary Information**

# Different scaling of linear models and deep learning in UKBiobank brain images versus machine-learning datasets

Schulz et al.

**Supplementary Figure 1**

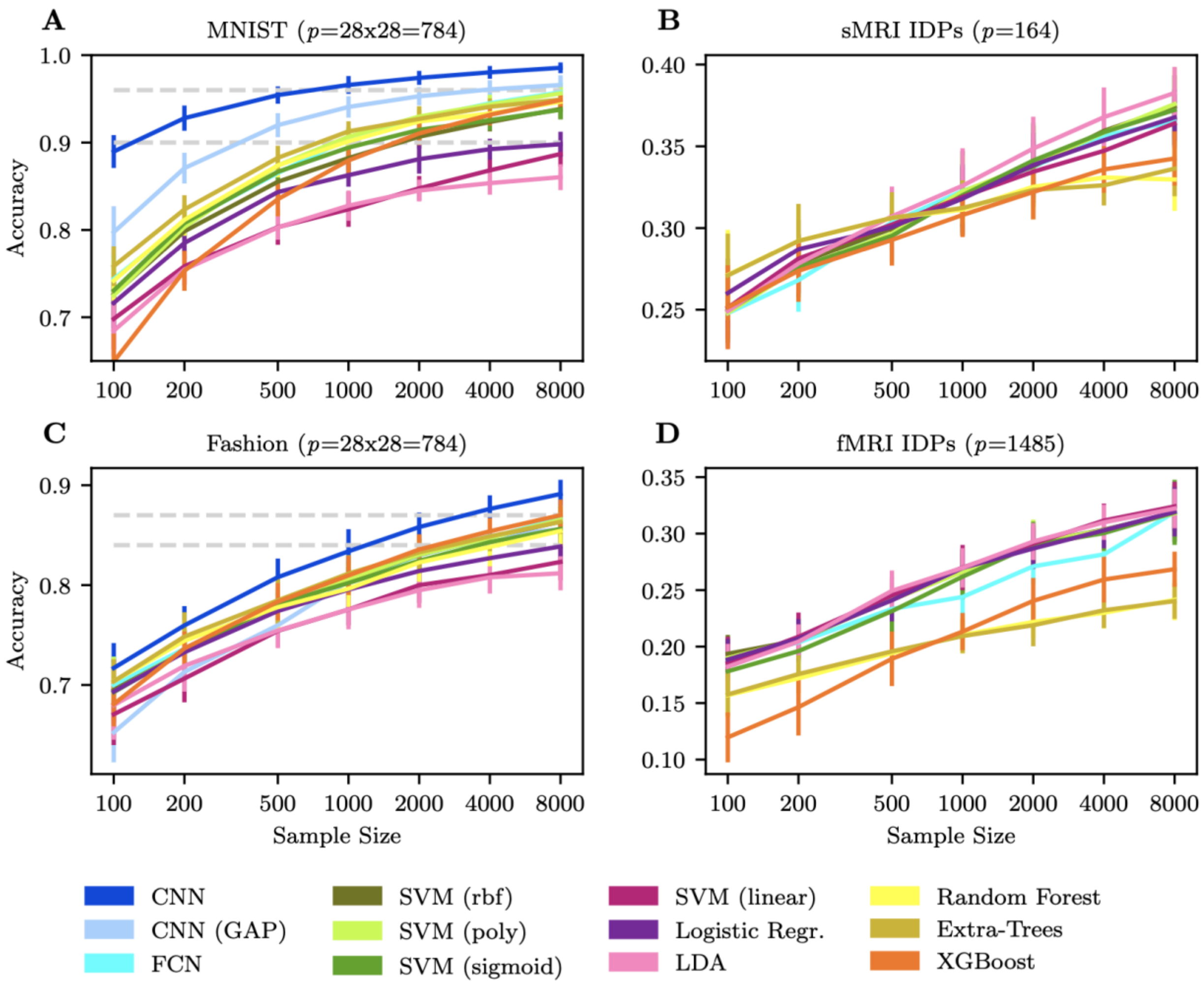


**Trees-based models show no evidence of exploitable nonlinear relationships in brain images.** The high-capacity estimators random forests (*1*), extremely randomized trees (*2*), and gradient boosted trees (*3*) achieved age/sex prediction performances similar to kernel models on the MNIST and Fashion images (A/C), but underperformed compared to linear and kernel models on brain images (B/D, IDP=image-derived phenotype). Due to the hyperparameter tuning, the examined kernel models approximate sample-efficient linear models in the absence of exploitable nonlinear relationships and thus performed like linear models on brain data. However, decision trees are generally acknowledged to be ill-suited to model linear relationships - having to approximate them by means of a step function. Their weak performance on brain data again suggests a primarily linear structure of the examined brain-imaging modalities. The number of input variables in a modeling scenario is denoted by *p.* Error bars = mean +/- 1 standard deviation across 20 cross-validation iterations (all panels).

**Supplementary Figure 2**

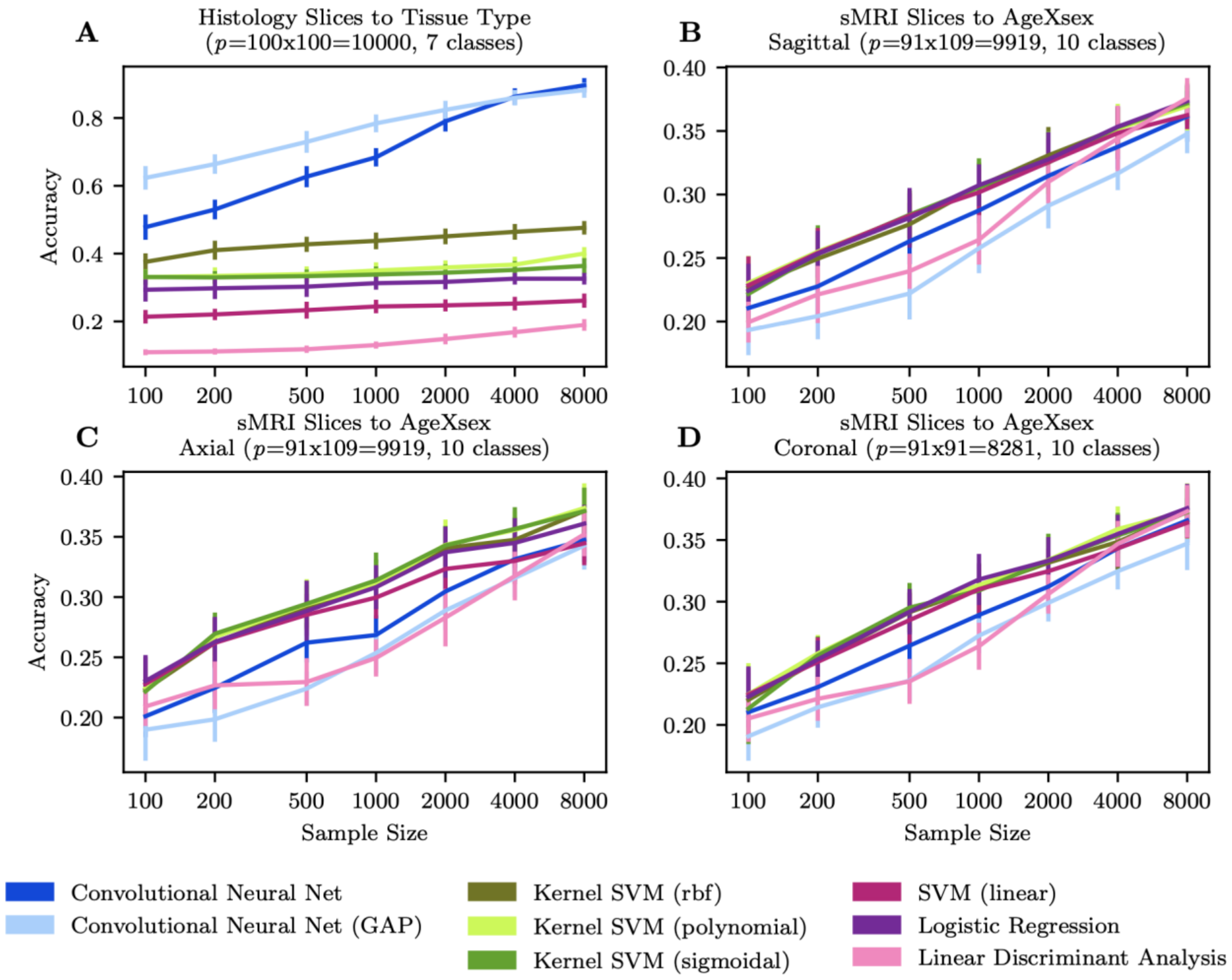


**Stained histological images of cancerous body tissue slices allow powerful predictions.** In addition to reference datasets from the machine learning community, we analyzed a recently available oncological dataset (*4*). In this alternative biomedical dataset, histology slices of healthy and cancerous body tissue are to be categorized into seven tissue classes. We transformed the tissue slice images to grayscale images (via relative luminance) and resized them by downsampling (via nearest neighbour resampling) the resolution to match our whole-brain sMRI slices. In the tissue images (A), we clearly observed the expected increase in prediction performance when applying more and more expressive models. These results serve as a positive test that our model implementations, especially our convolutional neural network architectures, did indeed work properly on 100x100 pixel data. This in turn suggests that the lack of exploitable nonlinear information in sMRI slices (B/C/D) for phenotype prediction is a property of brain-imaging data, rather than a problem of high dimensionality or hyperparameter tuning (cf. Supplementary Fig. 5 and 6). The number of input variables in a modeling scenario is denoted by *p.* Error bars = mean +/- 1 standard deviation across 20 cross-validation iterations (all panels).

**Supplementary Figure 3**

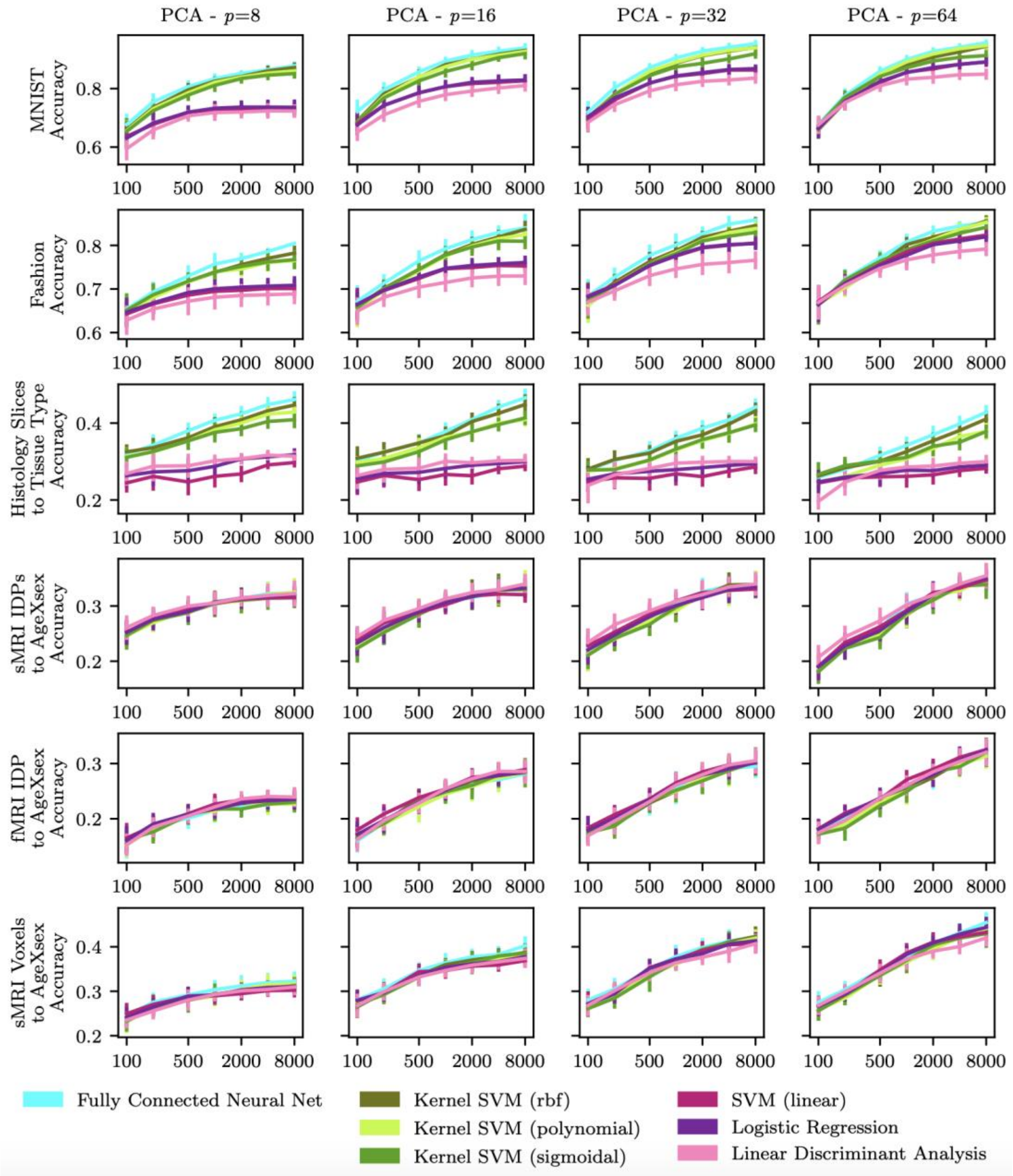


**Difference in feature dimensionality does not explain inability to exploit nonlinear structure.** After carrying out linear dimensionality reduction using principal component analysis (PCA), kernel and deep models clearly outperformed linear models in all examined reference datasets, but not in brain-imaging data. For MNIST, Fashion and histology slices, the performance gains from nonlinear models were more pronounced for lower-dimensional representations. In contrast, linear and nonlinear models performed indistinguishably on brain images. Moreover, model performance on MNIST, Fashion, and histology slices saturated in all PCA settings, while model performance on brain-imaging data saturated more slowly (latent embeddings $p<64$), or not at all (latent embeddings $p=64$). Repeating these analyses for univariate feature selection, recursive feature elimination, and random projections led to qualitatively similar results with the same conclusion. The number of input variables in a modeling scenario is denoted by *p.* Error bars = mean +/- 1 standard deviation across 20 cross-validation iterations (all panels).

**Supplementary Figure 4**

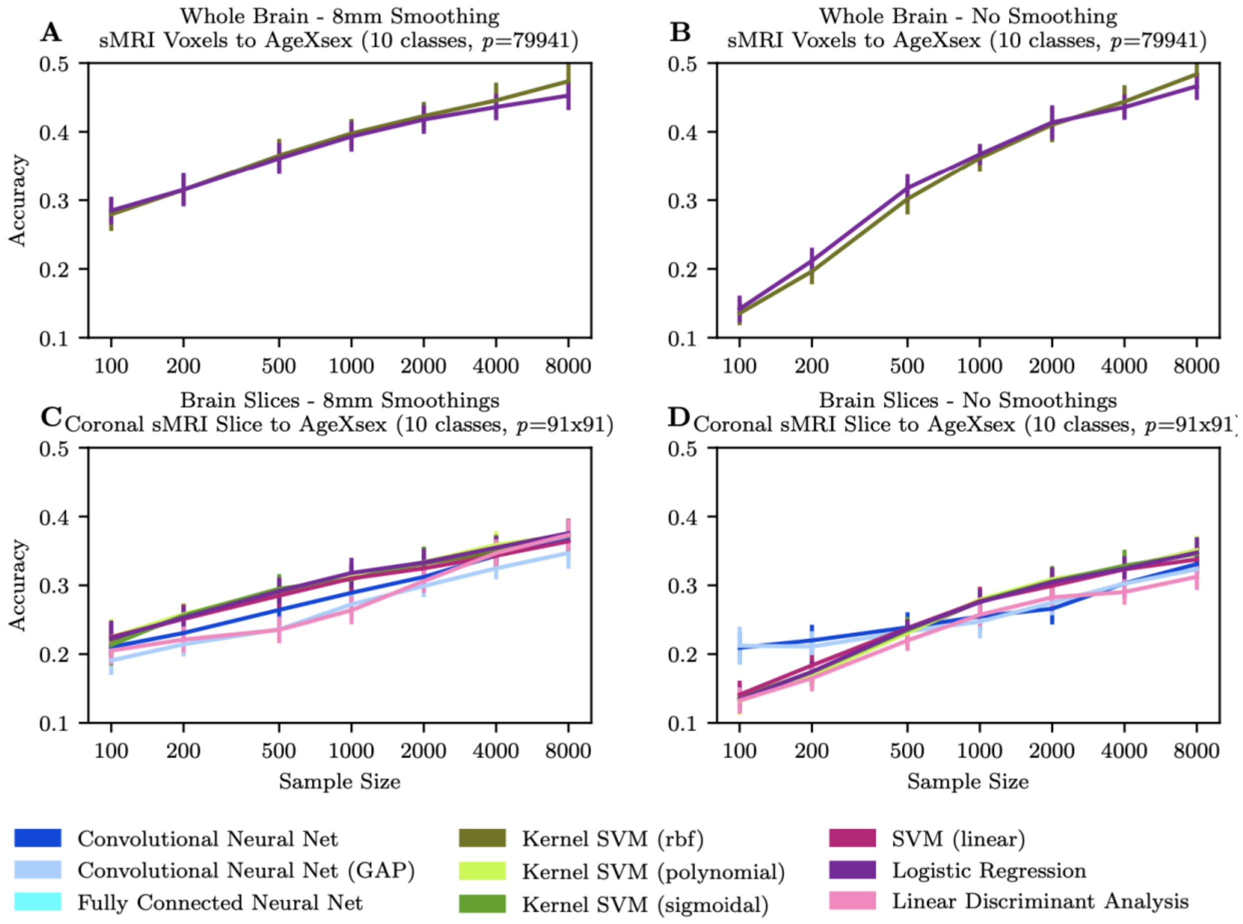


**Whole-brain volumes do not reveal evidence of exploitable nonlinear structure.** Even when using the full set of gray matter masked sMRI voxels, we observed no evidence of exploitable nonlinear relationships, as well as no clear saturation of prediction performance. This result provides additional evidence that our results obtained after a stage of feature selection are not an artifact of the applied feature reduction methods: Even without targeted feature selection, we observe no evidence of exploitable nonlinear structure in sMRI data for phenotype prediction. Moreover, Gaussian smoothing of voxels does not explain the lack of exploitable nonlinear relationships in sMRI data. Part of our preprocessing of raw sMRI voxels was the application of well-established 8mm Gaussian smoothing (A/C). We repeated our analysis without this smoothing step (B/D). All examined models performed better on smoothed data. On smoothed as well as non-smoothed data, deep and kernel models did not generally outperform simpler linear models. The number of input variables in a modeling scenario is denoted by *p.* Error bars = mean +/- 1 standard deviation across 20 cross-validation iterations (all panels).

## Supplementary Figure 5

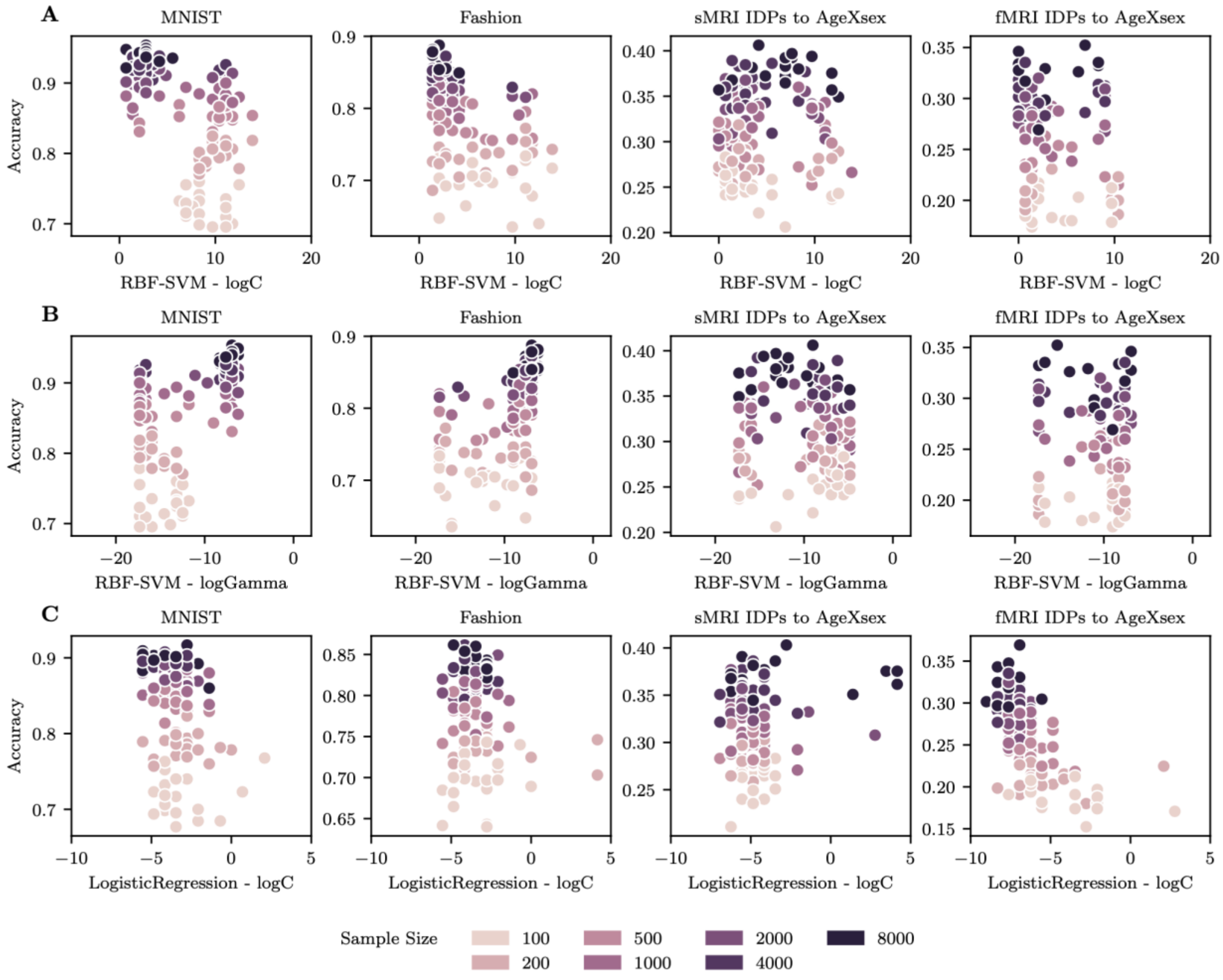


**Hyperparameter grids for model selection are sufficiently fine-grained.** Depicts the selected best hyperparameter combinations for each of 20 cross-validation subsampling repetitions (dots) across a range of training set sizes (hues). The grid of hyperparameters appears to have been sufficiently fine-grained, as a range of different hyperparameter combinations were found to be optimal for each sample size. Conversely, if all subsampling repetitions were to converge on the exact same hyperparameter combinations, this would indicate an inadequately coarse search grid. Shows results for RBF-SVM (figure rows A and B correspond to the relevant hyperparameters "C" and "gamma", respectively), the repeatedly best-performing kernel method, and logistic regression (C), the repeatedly best performing linear method. Results for the other models are qualitatively similar.

**Supplementary Figure 6**

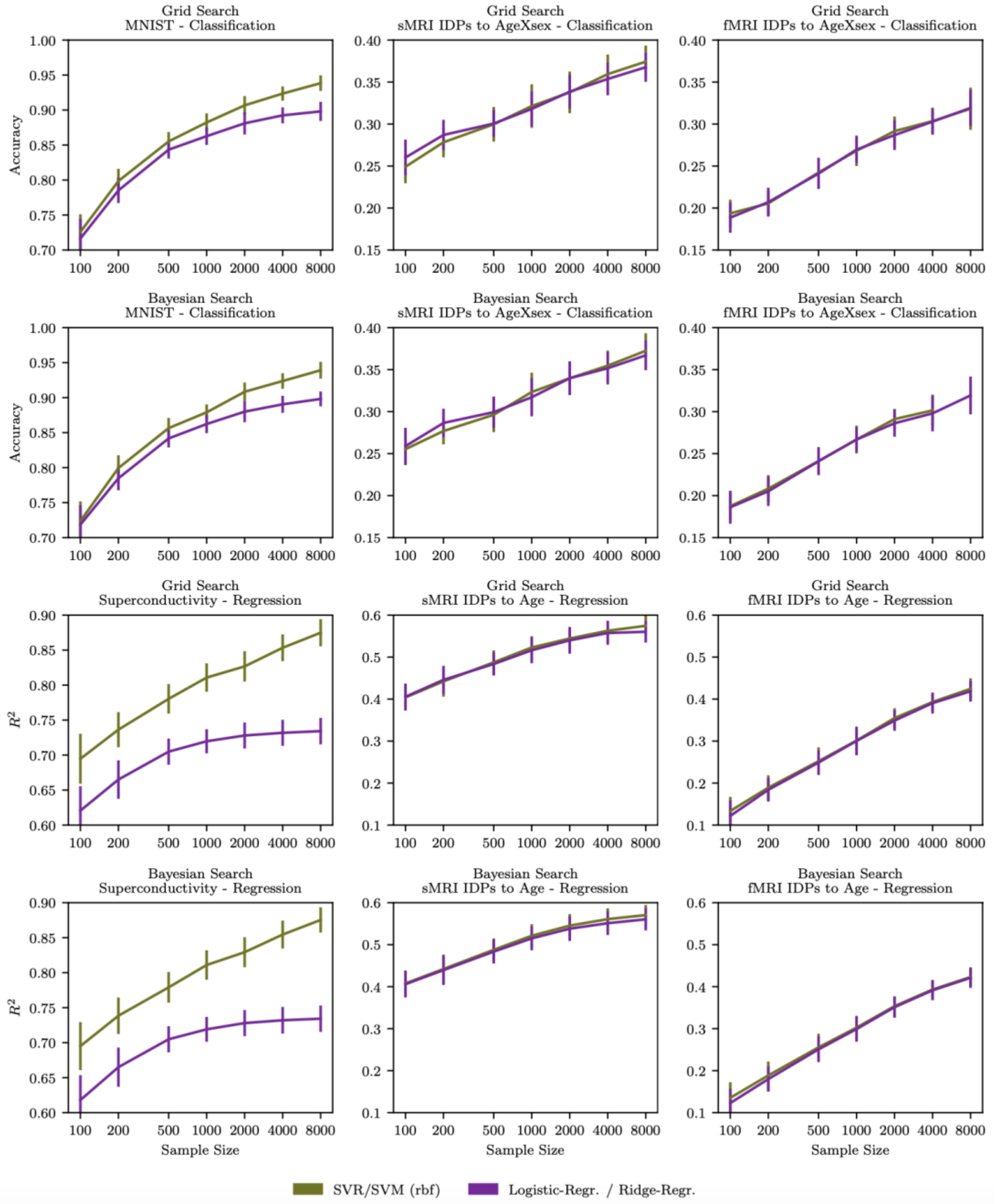


**Continuous adaptive Bayesian hyperparameter search leads to equivalent results.** For the strongest linear and kernel models (logistic regression and RBF-SVM), analyses were repeated with Bayesian hyperparameter search (*5*) and 500 evaluations per search. Results were consistent between smooth Bayesian hyperparameter search and grid search with discrete candidate hyperparameters. These supplementary analyses underline that our observations are unlikely to be a result of insufficient hyperparameter tuning. IDP=image-derived phenotype. Error bars = mean +/- 1 standard deviation across iterations (all panels).

## Supplementary References

1. Breiman, L. (2001). Random forests. Machine learning 45 (1), 5-32.
2. Geurts, P., Ernst, D., & Wehenkel, L. (2006). Extremely randomized trees. Machine learning, 63(1), 3-42.
3. Friedman, J. H. (2001). Greedy function approximation: a gradient boosting machine. Annals of statistics, 1189-1232.
4. Kather, Jakob Nikolas, Alexander T. Pearson, Niels Halama, Dirk Jäger, Jeremias Krause, Sven H. Loosen, Alexander Marx, et al. (2019). Deep Learning Can Predict Microsatellite Instability Directly from Histology in Gastrointestinal Cancer. Nature Medicine 25 (7): 1054–56.
5. Bergstra, J., Yamins, D., & Cox, D. D. (2013). Making a science of model search: Hyperparameter optimization in hundreds of dimensions for vision architectures.
